# Supplementary material for: Colorectal Tumour Mucosa Microbiome Is Enriched in Oral Pathogens and Defines Three Subtypes That Correlate with Markers of Tumour Progression
Source: Cancers (Basel). 2021 Sep 25;13(19):4799. doi: 10.3390/cancers13194799 (PMC8507728; doi:10.3390/cancers13194799)
Supplement: Supplementary file 1 [file cancers-13-04799-s001.zip › cancers-1377747-supplementary-updated final/cancers-1377747-supplementary.pdf]

Supplementary Materials

Colorectal Tumour Mucosa Microbiome Is Enriched in Oral Pathogens and Defines Three Subtypes That Correlate with Markers of Tumour Progression

Barbora Zwinsová, Vyacheslav A. Petrov, Martina Hrivňáková, Stanislav Smatana, Lenka Micenková, Natálie Kazdová, Vlad Popovici, Roman Hrstka, Roman Šefr, Beatrix Bencsiková, Lenka Zdražilová-Dubská, Veronika Brychtová, Rudolf Nenutil, Petra Vídeňská and Eva Budinská

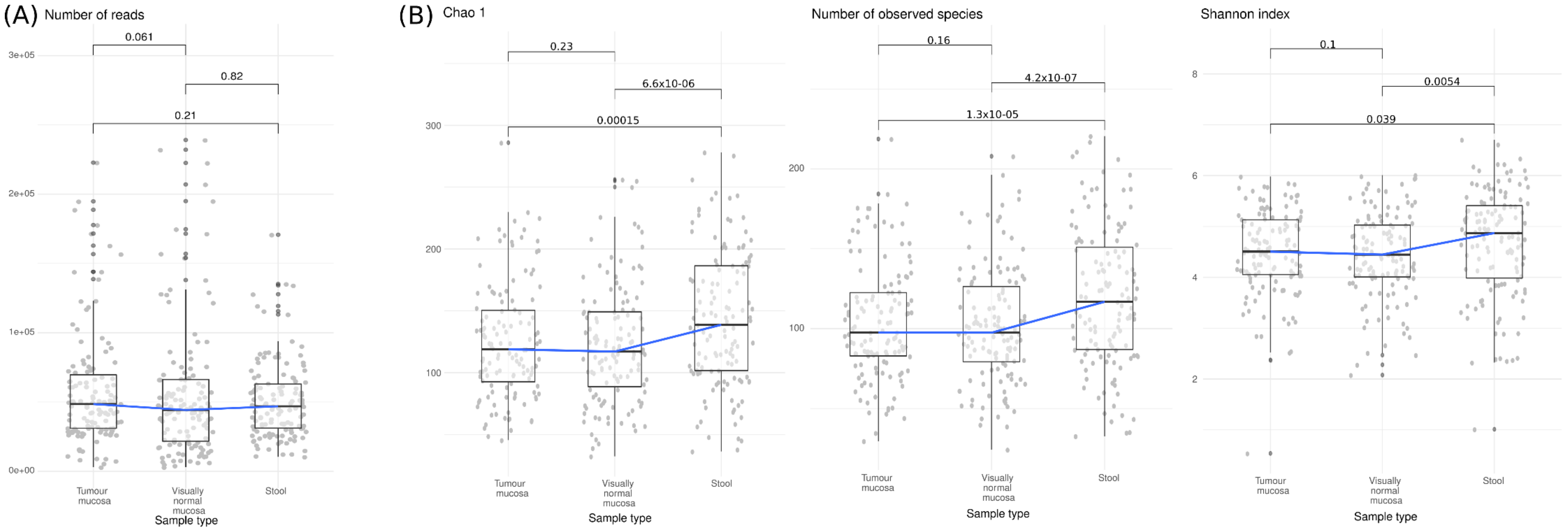

Figure S1. Summary statistics of (**A**) number of reads and (**B**) diversity comparison of three sample types on 127 triplets. The boxplot middle vertical line represents median, the box represents the interquartile range (IQR), the whiskers extend to +/−1.5 IQR. The dark grey dots represent outlier values (outside the whiskers region). The blue line connects medians, grey dots represent scattered original data points. The numbers over the boxplots represent *p*-values from pairwise statistical testing.

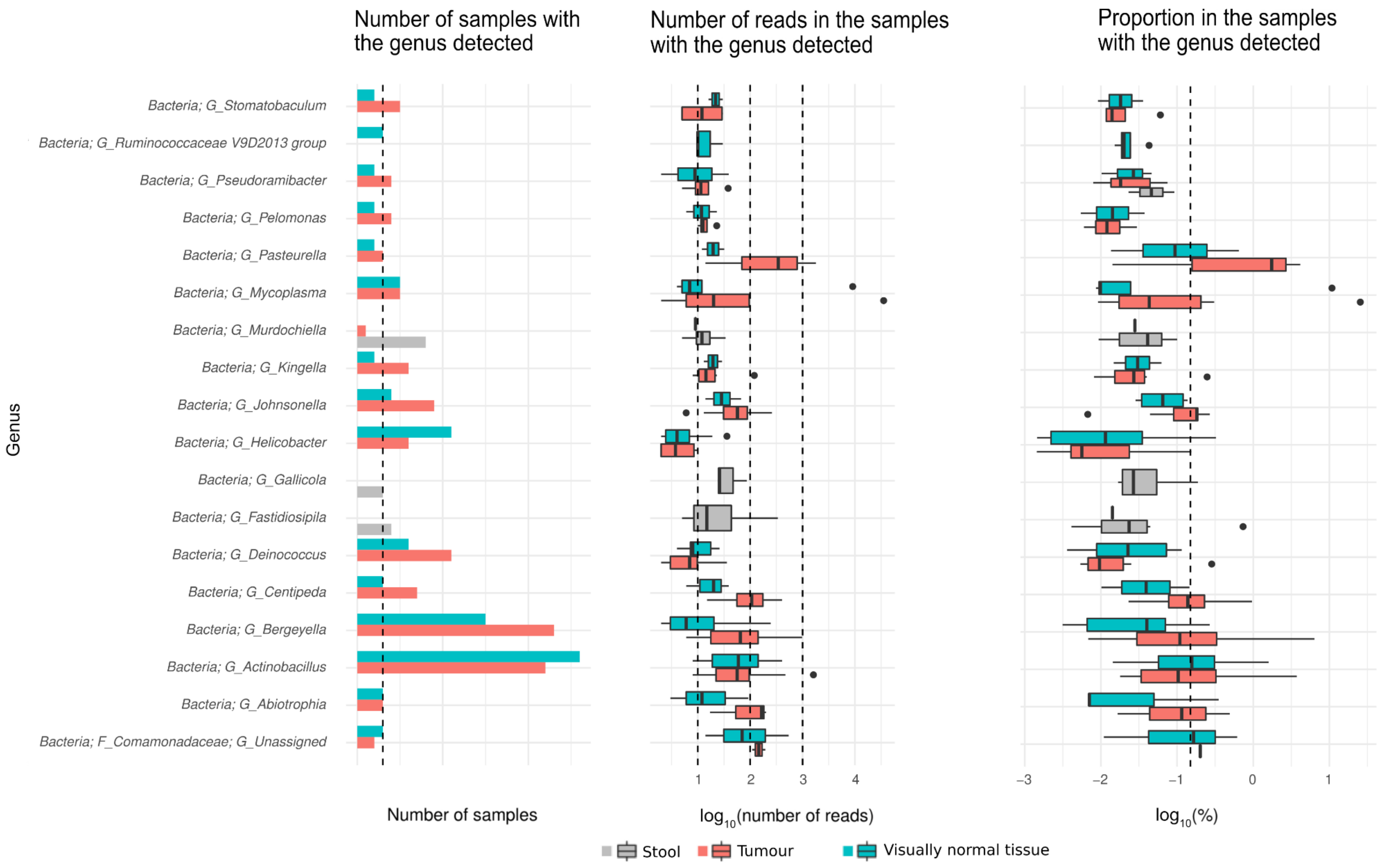

Figure S2. Summary statistics of incidence and abundance (absolute and relative) of the genera which were detected only in some of the three sample types. The boxplot middle vertical line represents median, the box represents the interquartile range (IQR), the whiskers extend to +/−1.5 IQR. The black dots represent outlier values (outside the whiskers region). The dashed vertical lines represent: (i) left: a threshold value of three samples with the genus detected, (ii) middle: thresholds of 10, 100 and 1000 reads and (iii) median relative abundance value of all genera in the samples with the genus detected.

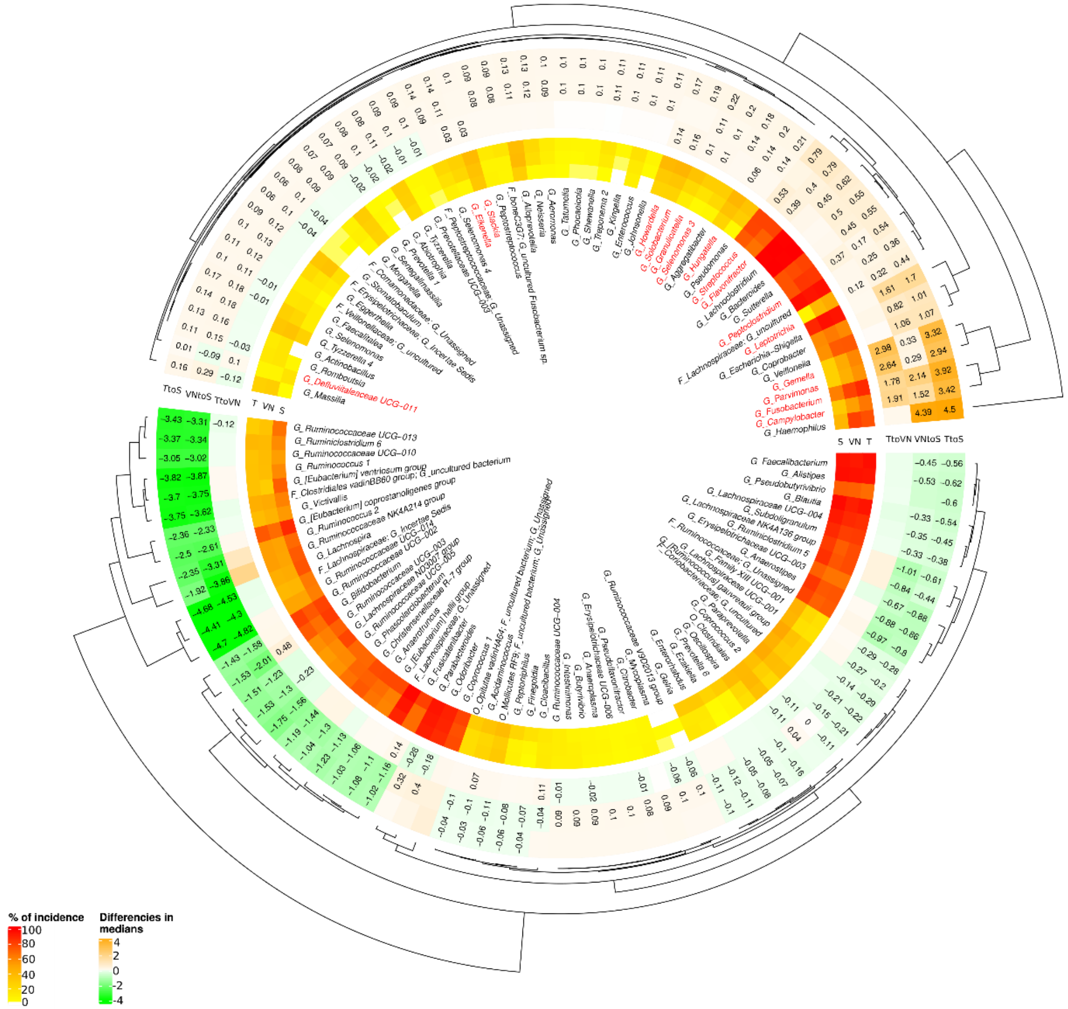

Figure S3. Differences in microbiome composition across the sample types visualized in a circular heatmap of the proportion and incidence of the significant genera The graph shows two heatmaps with dendrograms—results of clustering of tumour genera (top semi-circle) and all other significant genera (bottom semi-circle). Red font demarks tumour specific genera. T—tumour swabs, VN—visually normal mucosa swabs, S—stool, G—genus, F—family.

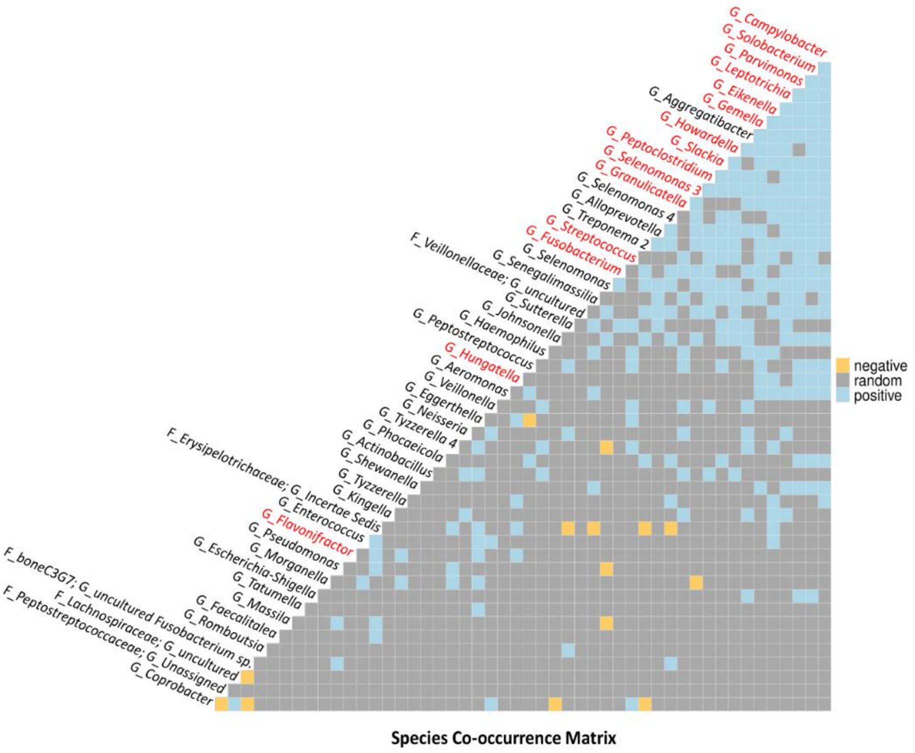

Figure S4. Co-occurrence analysis of 57 tumour genera, red colour demarks tumour specific genera. (G—genus, F—family, sp—species).

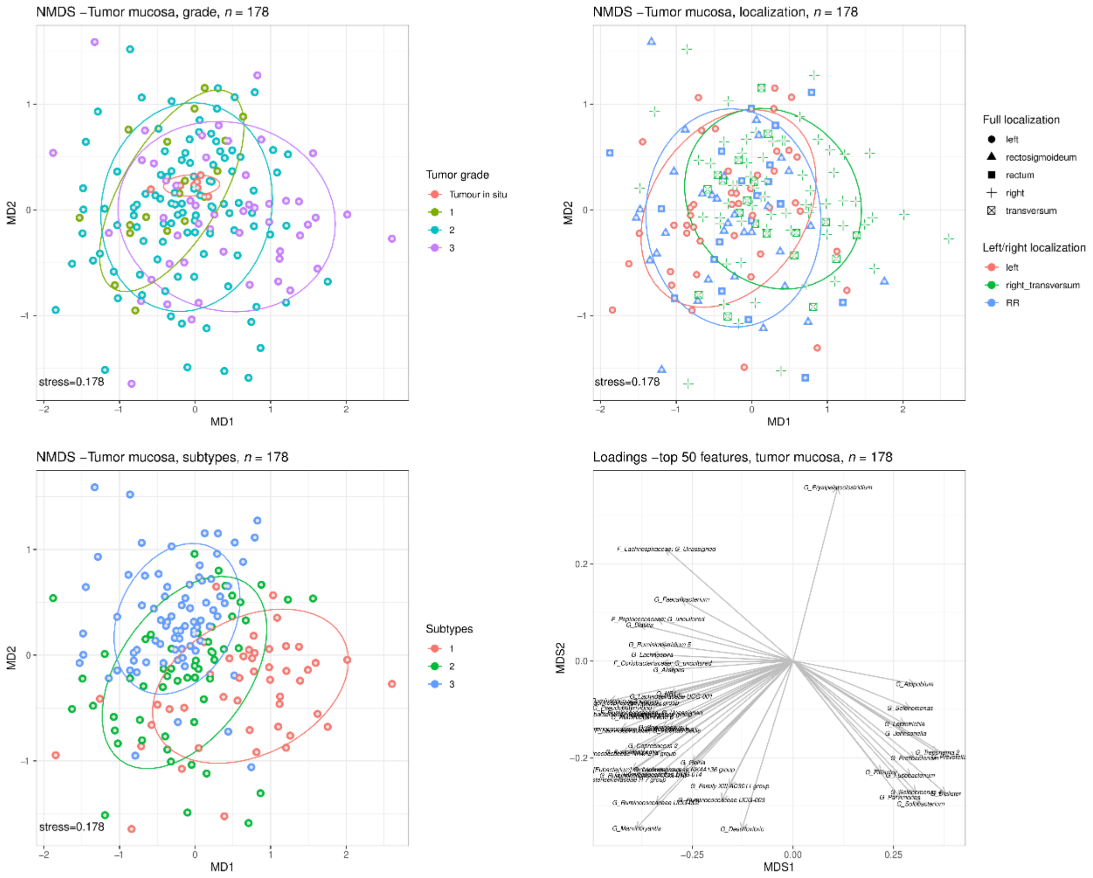

Figure S5. Microbial β-diversity analysis by NMDS on 178 tumour tissue swab. The graphs show probability ellipses for each plotted group at level 0.8. (G—genus, F—family).

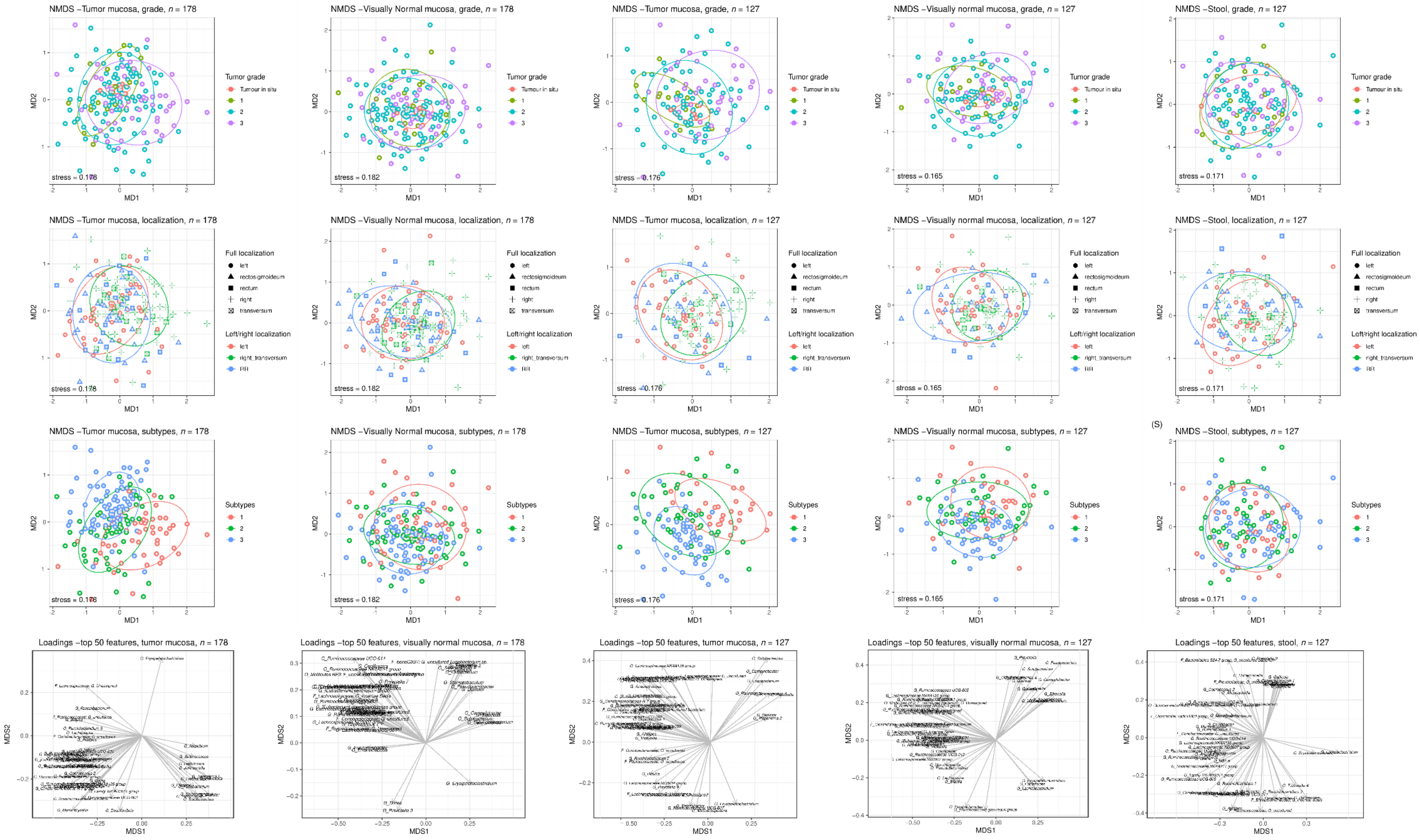

Figure S6. Microbial β-diversity analysis by NMDS performed on 127 triplets. The graph shows probability ellipses for each plotted group, level = 0.8.

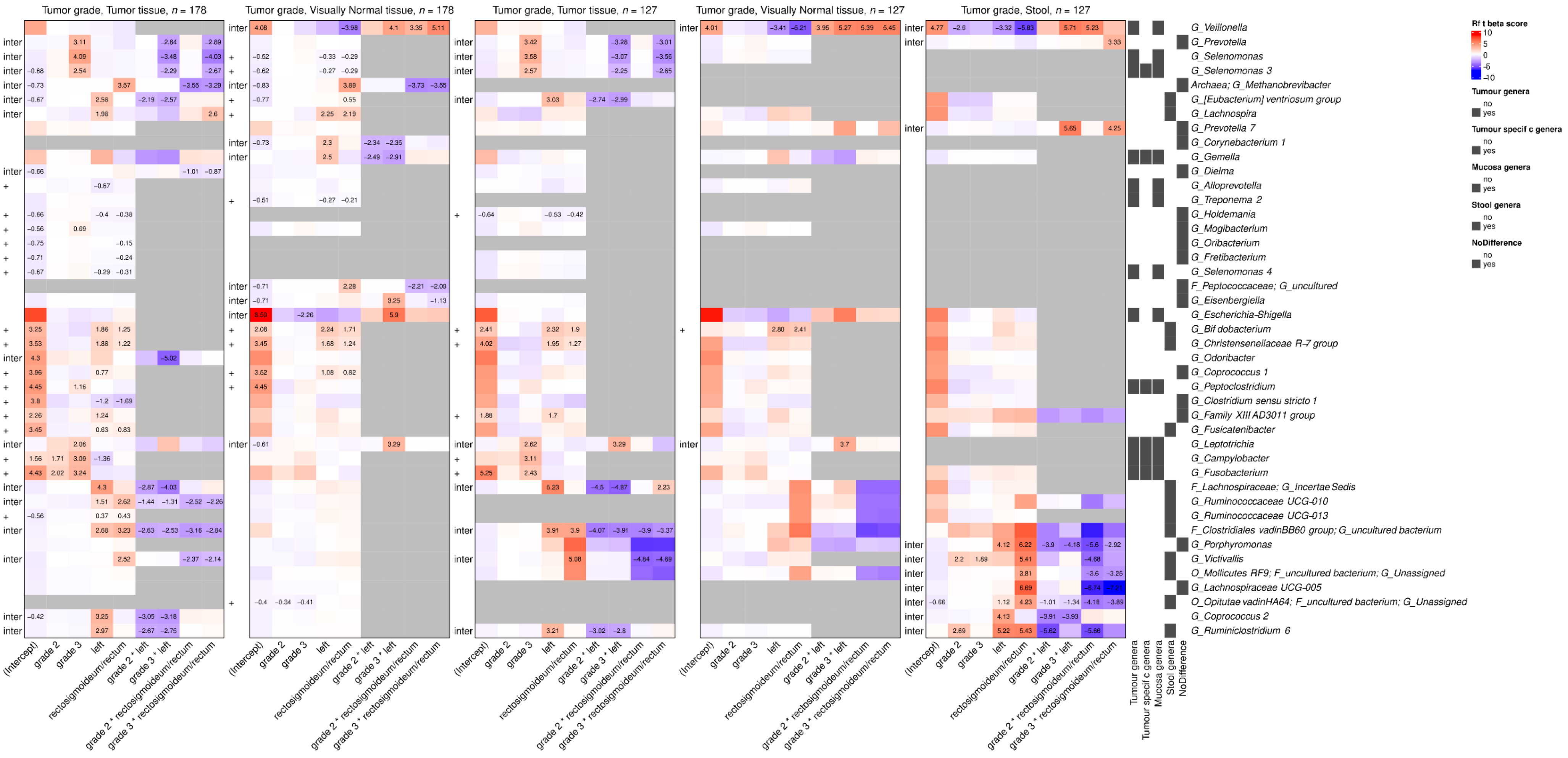

Figure S7. Side-dependent associations between tumour histological grade and microbiota composition. (G—genus, F—family, O—order, inter-interaction model used, + —model without interaction)

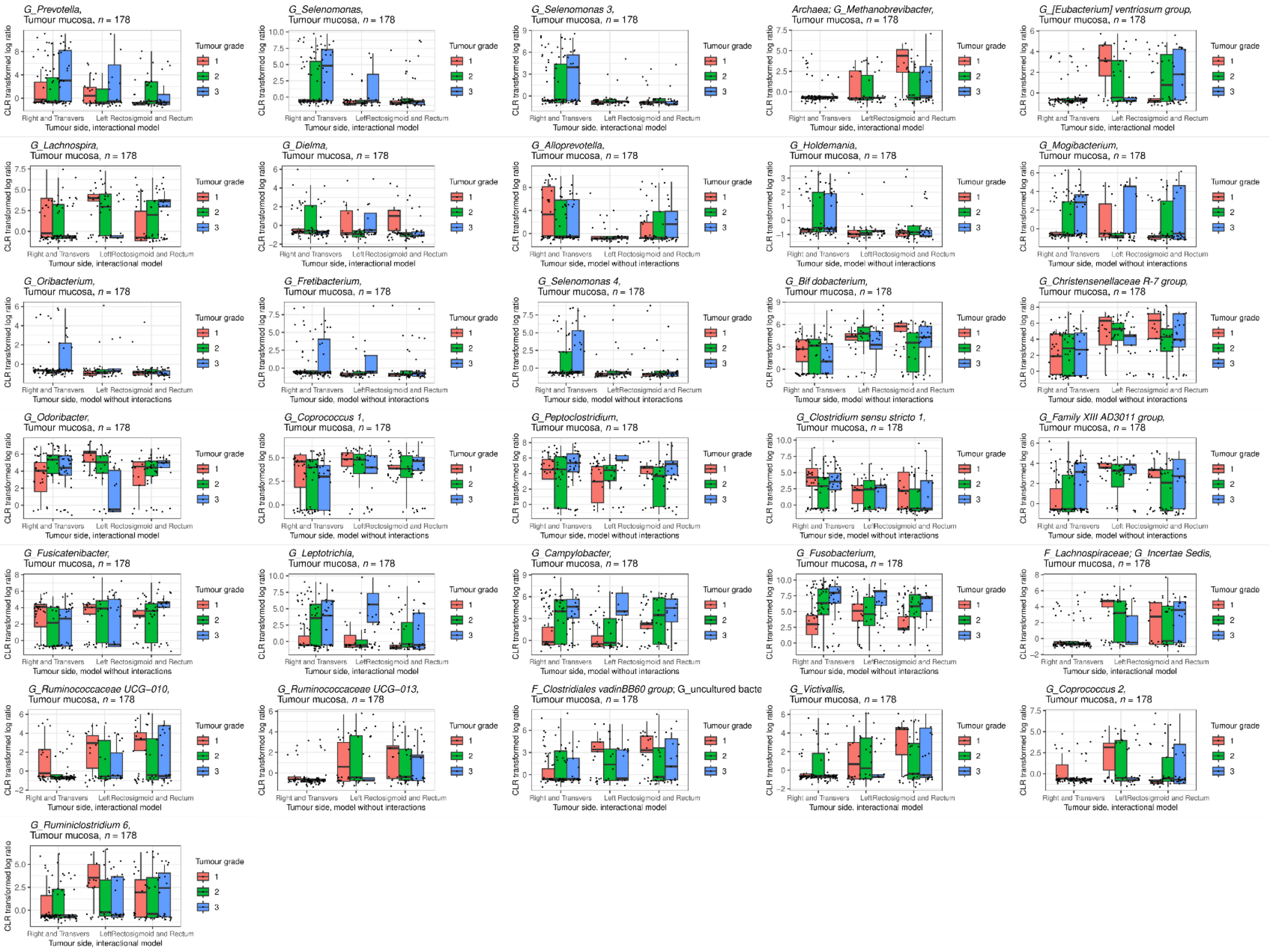

Figure S8. Boxplots of distribution of clr transformed abundance of genera associated with tumour grade and/or location in 178 tumour mucosa samples in models with or without interaction at *p*-value < 0.05. The boxplot middle vertical line represents median, the box represents the interquartile range (IQR), the whiskers extend to +/−1.5 IQR. The black dots represent individual samples. (G—genus, F—family, clr—centered log-ratio transformation)

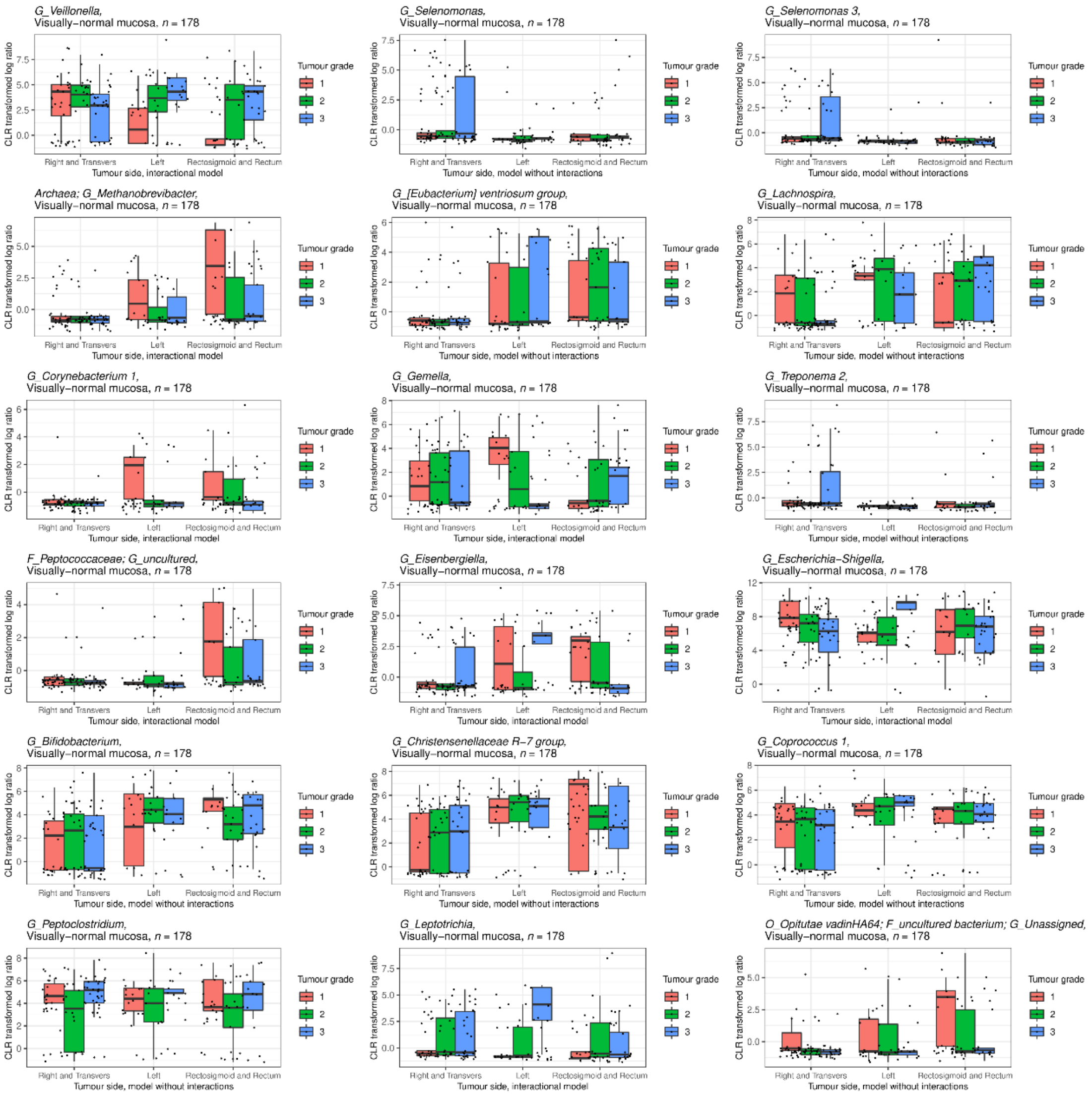

Figure S9. Boxplots of distribution of clr transformed abundance of genera associated with tumour grade and/or location in 178 visually normal mucosa samples in models with or without interaction at p-value < 0.05. The boxplot middle vertical line represents median, the box represents the interquartile range (IQR), the whiskers extend to +/−1.5 IQR. The black dots represent individual samples. (G—genus, F—family, O—order, clr—centered log-ratio transformation)

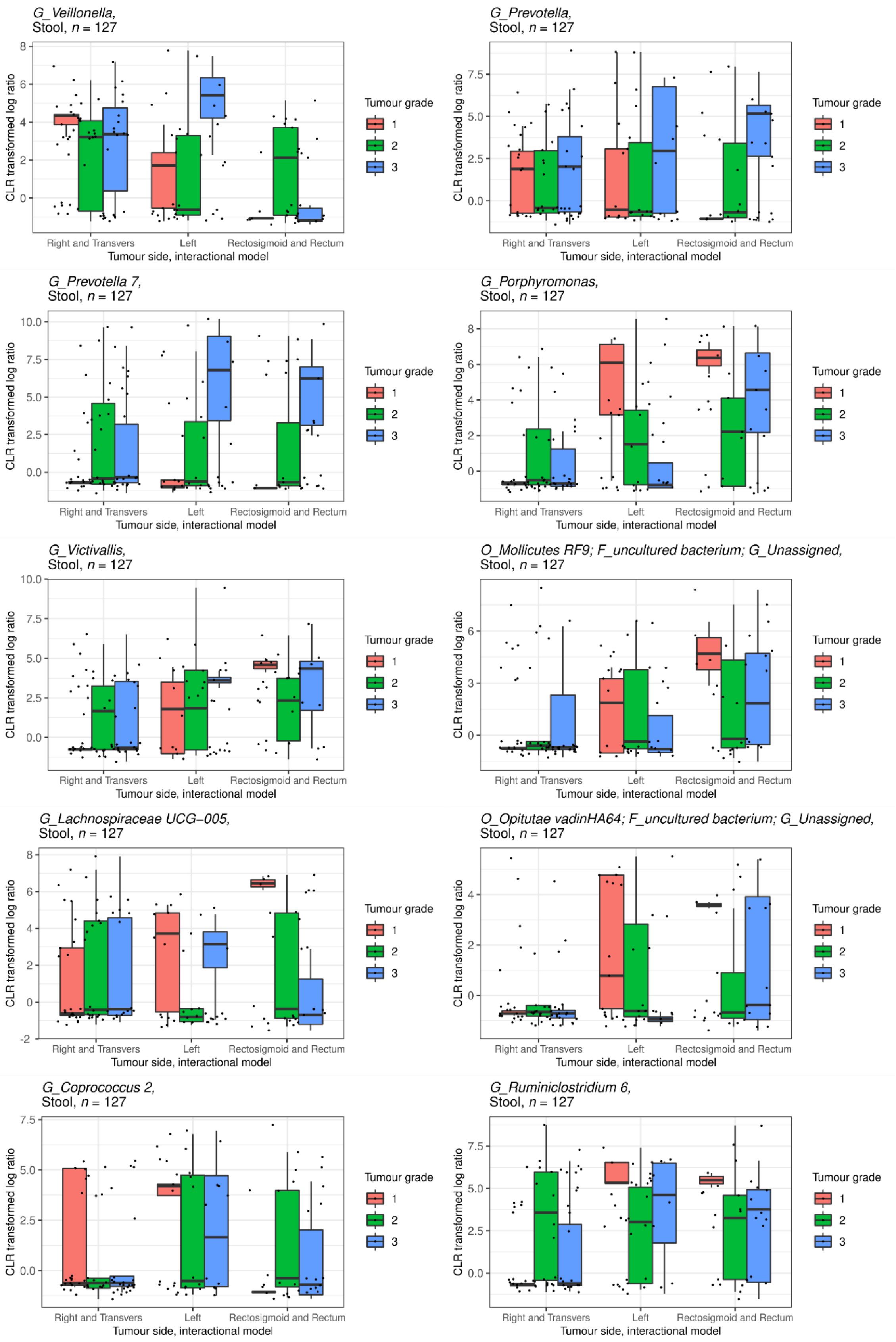

Figure S10. Boxplots of distribution of clr transformed abundance of genera associated with tumour grade and/or location in 127 stool samples in models with or without interaction at *p*-value < 0.05. The boxplot middle vertical line represents median, the box represents the interquartile range (IQR), the whiskers extend to +/−1.5 IQR. The black dots represent individual samples. (G—genus, F—family, O—order, clr—centered log-ratio transformation)

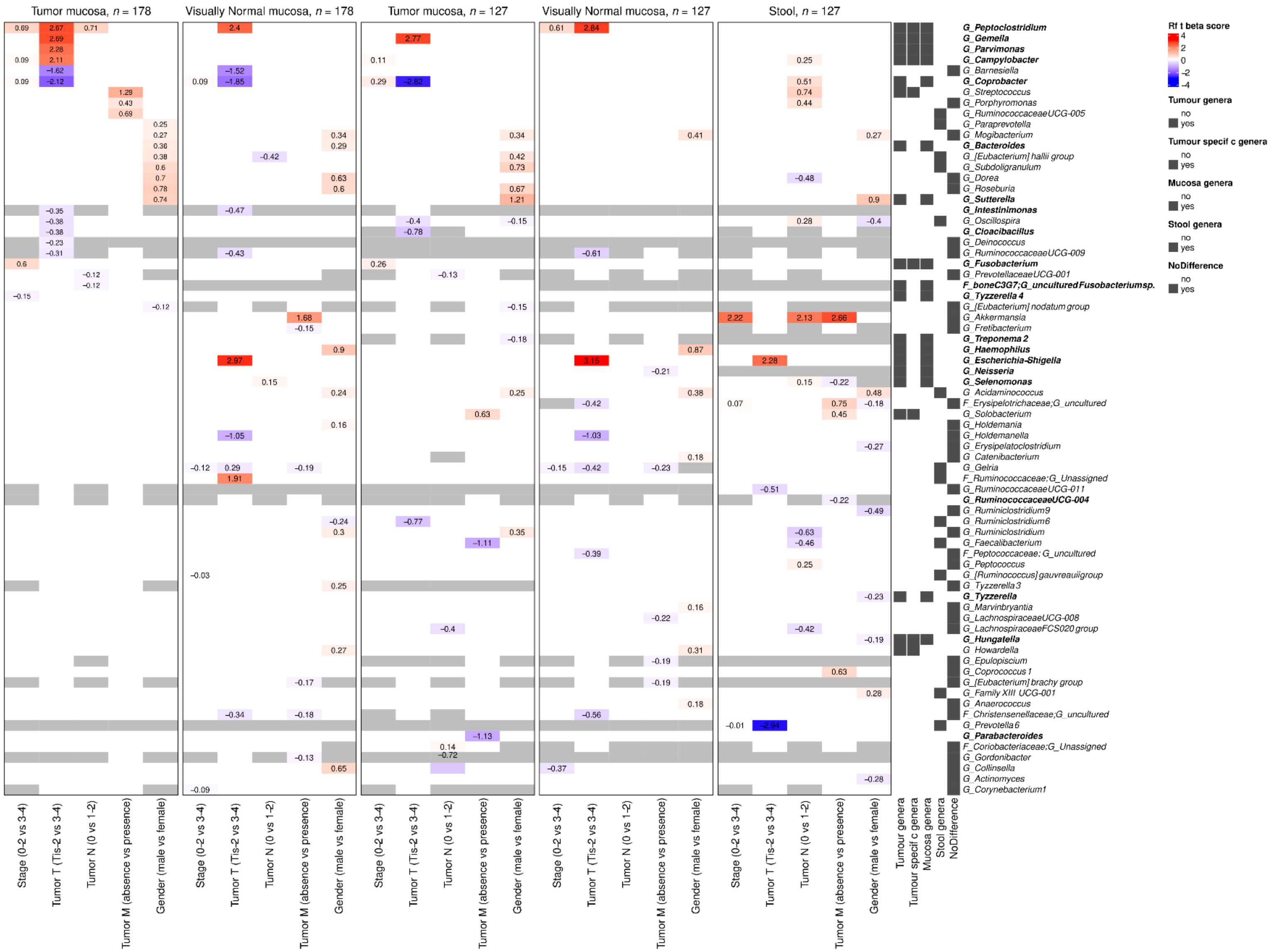

Figure S11. Associations between tumour stage, including TNM staging separately, gender and microbiota composition in all sample types and sample sizes. (G—genus, F—family, O—order, T—tumour pathologic stage, N—regional lymph nodes pathologic stage, M—synchronous distant metastasis)

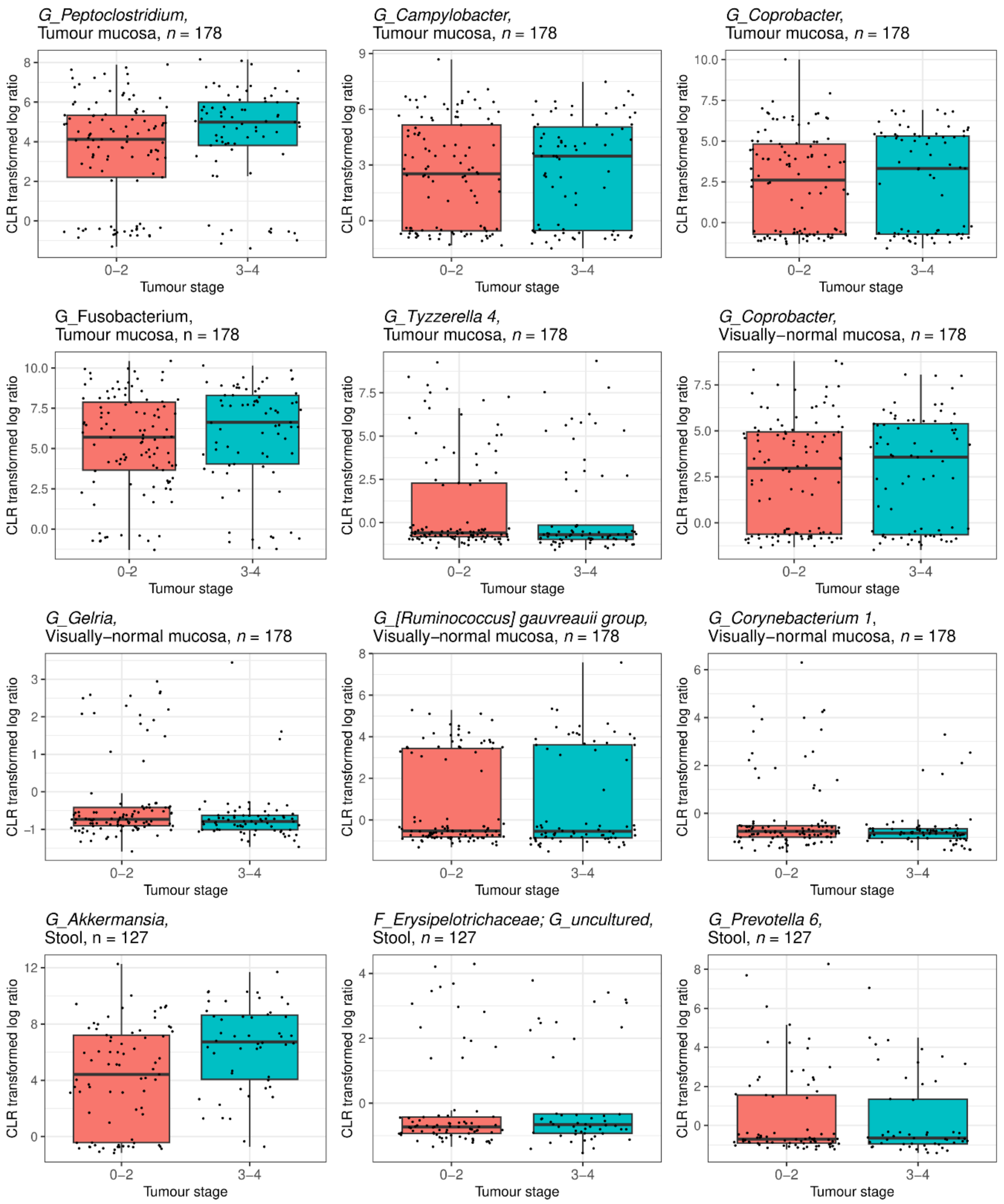

Figure S12. Boxplots of distribution of clr transformed abundance of genera associated with tumour stage in 178 tumour mucosa samples, 178 adjacent visually normal mucosa samples and 127 stool samples at *p*-value < 0.05. The boxplot middle vertical line represents median, the box represents the interquartile range (IQR), the whiskers extend to +/−1.5 IQR. The black dots represent individual samples. (G—genus, F—family, clr—centered log-ratio transformation)

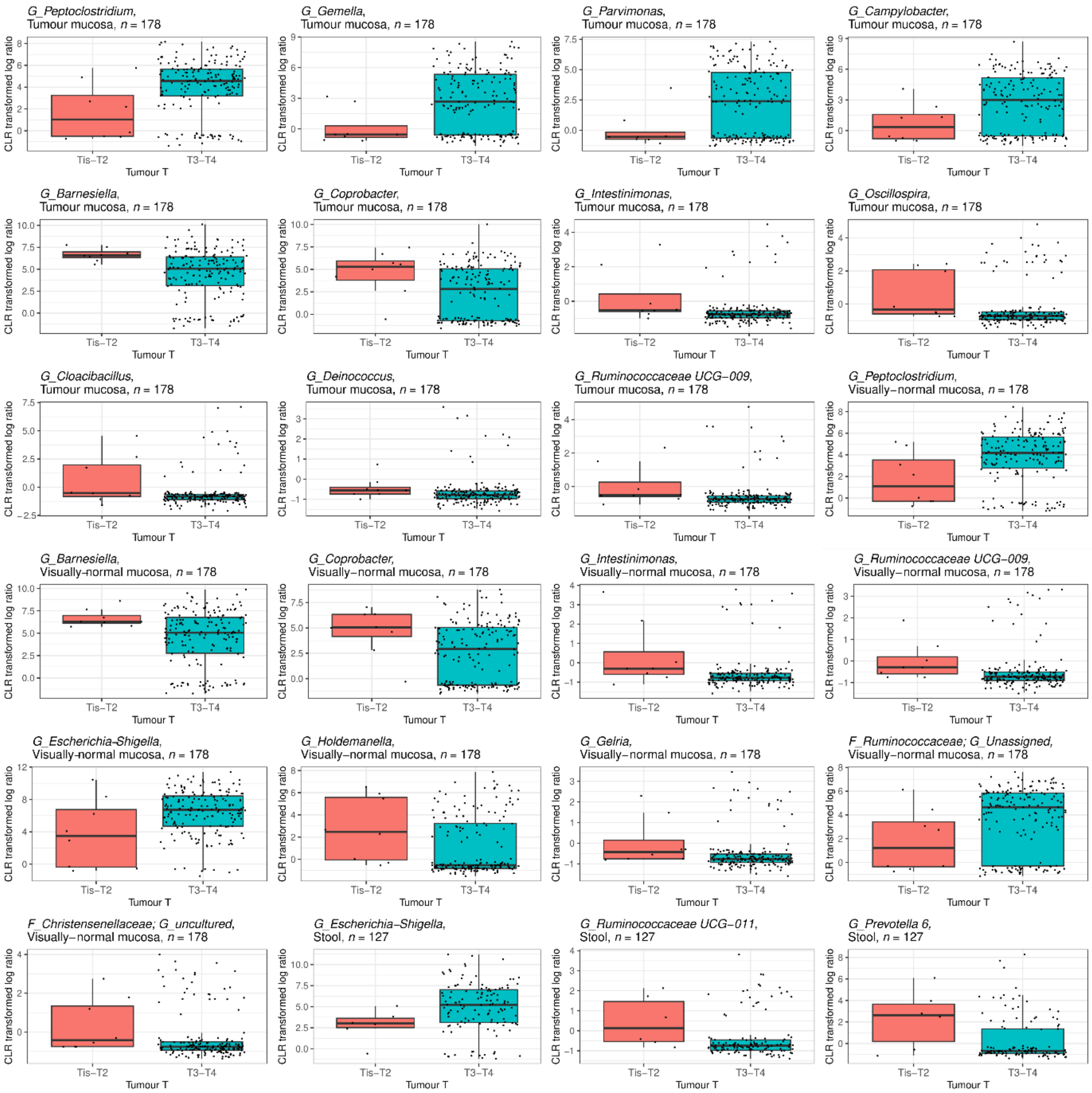

Figure S13. Boxplots of distribution of clr transformed abundance of genera associated with tumour pathologic stage in 178 tumour mucosa samples, 178 adjacent visually normal mucosa samples and 127 stool samples at *p*-value < 0.05. The boxplot middle vertical line represents median, the box represents the interquartile range (IQR), the whiskers extend to +/−1.5 IQR. The black dots represent individual samples. (G—genus, F—family, clr—centered log-ratio transformation, T—tumour pathologic stage)

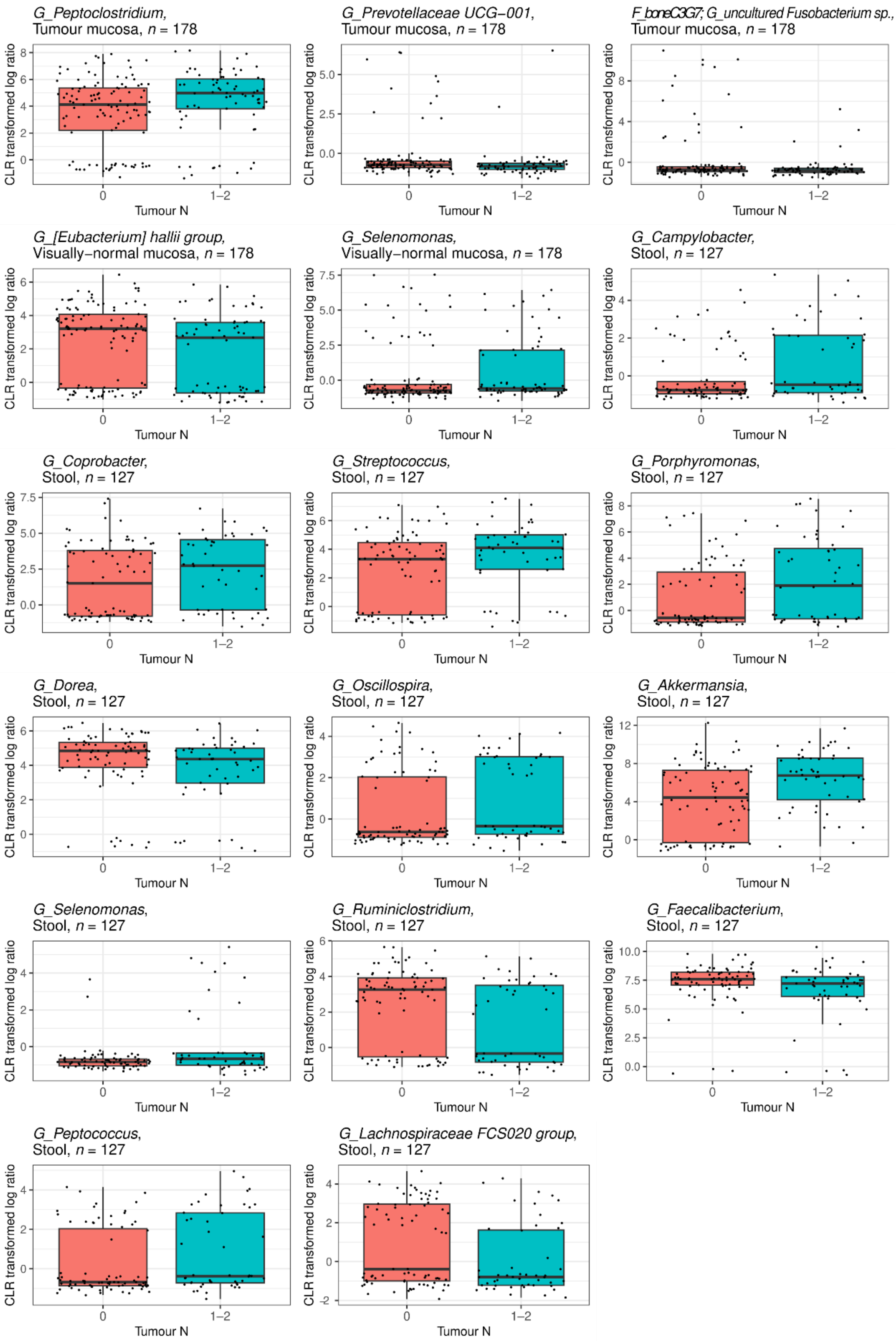

Figure S14. Boxplots of distribution of clr trans-formed abundance of genera associated with the presence of lymph-node metastases in 178 tumour mucosa samples, 178 adjacent visually normal mucosa samples and 127 stool samples at p-value < 0.05. The boxplot middle vertical line represents median, the box represents the interquartile range (IQR), the whiskers extend to +/−1.5 IQR. The black dots represent individual samples. (G—genus, F—family, clr—centered log-ratio transformation, N—regional lymph nodes pathologic stage)

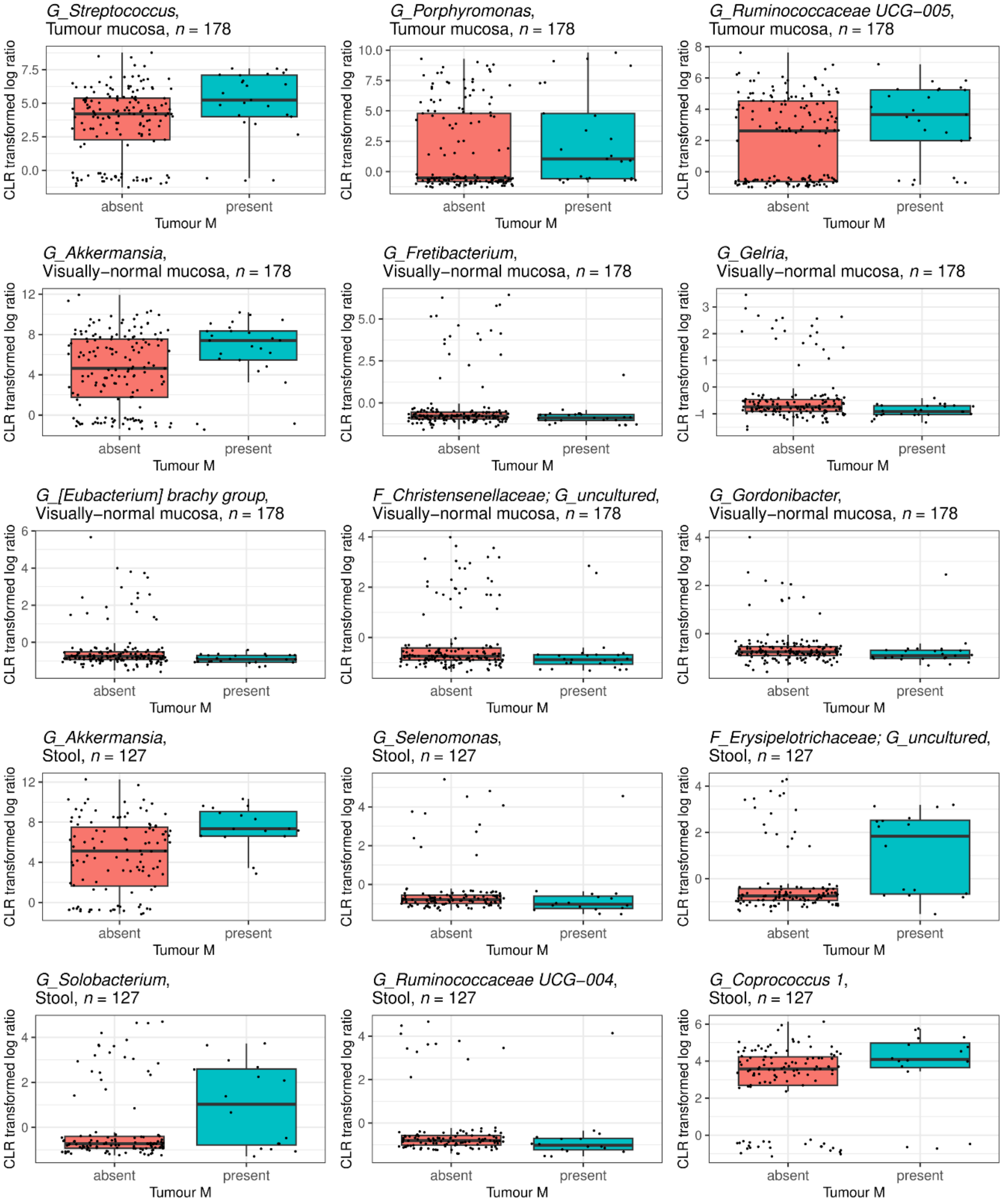

Figure S15. Boxplots of distribution of clr transformed abundance of genera associated with the presence of distance metastases in 178 tumour mucosa samples, 178 adjacent visually normal mucosa samples and 127 stool samples at *p*-value < 0.05. The boxplot middle vertical line represents median, the box represents the interquartile range (IQR), the whiskers extend to +/−1.5 IQR. The black dots represent individual samples. (G—genus, F—family, clr—centered log-ratio transformation, M—synchronous distant metastasis)

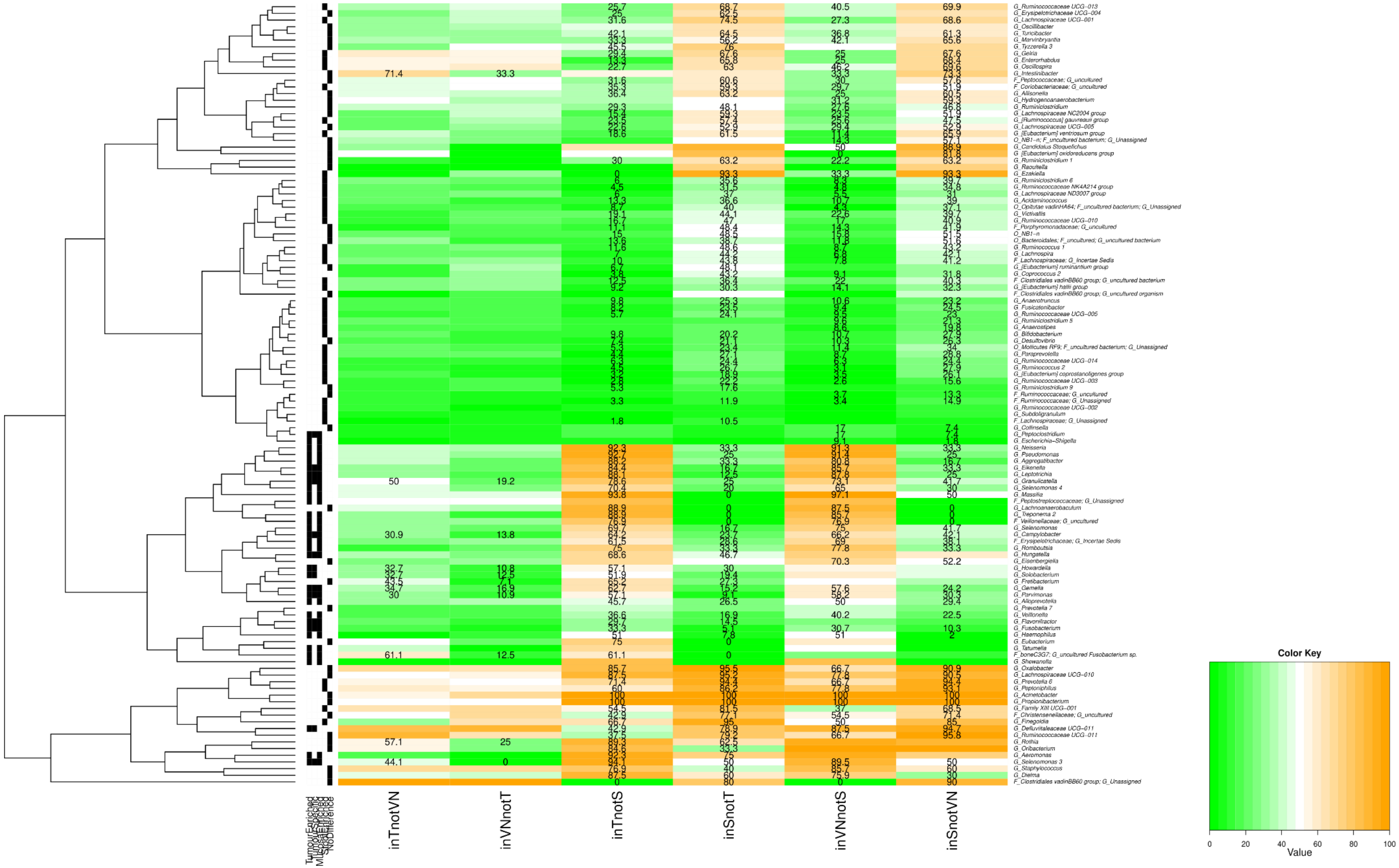

Figure S16. Results of pairwise coincidence analysis of genera across sample types within the same patient.

Text S1: Extended Results of Differences in Microbiome Diversity and Incidence Across the Sample Type

We were investigating the differences in microbiome abundance, incidence (presence/absence) and diversity across the different sample types. To reach this aim we analysed 483 samples (tumour tissue swabs, visually normal tissue swabs and stool samples) from 178 patients with stage 0–IV colorectal cancer by 16S rRNA sequencing. From 127 patients, all three sample types were available (called triplets) and 51 patients with mucosa duplets (both mucosal swabs). Even though the analysis was performed at all taxonomical levels, we mainly operated on lower taxonomical levels due to low ability of the 16S rRNA analysis to reliably assign the species.

1.1. Incidence Across Sample Types

In our study we observed changes in unique presence of some genera in sample types. 14 genera (*Stomatobaculum*, *Pseudoramibacter*, *Pelomonas*, *Pasteurella*, *Mycoplasma*, *Kingella*, *Johnsonella*, *Helicobacter*, *Deinococcus*, *Centipeda*, *Bergeyella*, *Actinobacillus*, *Abiotrophia* and one unassigned genus from order *Comamonadaceae*) representing five orders (*Mycoplasmatales, Deinococcales, Oceanospirillales, Frankiales* and *Sphingomonadales*) and five families (*Geodermatophilaceae*, *Deinococcaceae*, *Helicobacteraceae*, *Mycoplasmataceae*, *Sphingomonadaceae*) were detected only in mucosal (tumour and visually normal) samples (Figure S2). Two genera *Fastidiosipila* and *Gallicola* were detected only in stool samples, one genera *Murdochiella* was detected only in stool and tumour samples and one genera *Ruminococcaceae V9D2013* group was found only in visually normal samples.

In the next step, we focused on the analysis of incidence and abundance across sample types. Mainly between mucosal samples and stool we observed great differences in overall and pairwise comparison.

Phyla *Fusobacteria, Spirochaetae, Saccharibacteria* and *Deinococcus-Thermus* were found more often in mucosal samples compared to stool samples. For instance, *Fusobacteria* was found in 90.5% of tumour mucosa samples, 82.6% of visually normal mucosa samples and only in 65.4% of stool samples. On the contrary, phyla *Lentisphaerae* and *Tenericutes* were observed more often in the stool samples (Figure 2A last panel main manuscript).

We further analysed the pairwise incidence of the 264 genera across sample types using Cochran's Q test and subsequent pairwise McNemar's tests and found that 104 genera varied significantly across sample types (analysis of 127 triplets). Only 11 genera differed in incidence between tumour mucosa and visually normal samples, 101 genera between tumour and stool samples and 100 genera between visually normal and stool samples (with 84 differing between stool and both mucosa samples). Naturally, the increased incidence in some sample types correlated with their increased abundance (see below). We report the results as the co-occurrence of specific event pairs: genera present in one sample type but not in the second sample type and vice versa (Table S7, Figure S16, Figure 2A, fourth panel main manuscript).

1.2. Microbial Categorisation According to Sample Type

We compared microbial abundance across sample types on the genus level using Friedman rank sum test, to categorise the observed genera and interpret their possible origin on the tumour mucosa. Overall, of the 264 genera of *Bacteria* and *Archaea,* we found 121 genera with different expression between sample types (Table 2, Figure 1 main manuscript) and based on the results we defined five microbial categories.

57 genera were significantly more abundant in tumour mucosa compared to stool (Table 2, Figure 1, Figure 2 main manuscript). This group represents the category of core tumour genera and contains six of the above mentioned 14 mucosa-unique genera (present only in mucosal samples), namely *Stomatobaculum, Kingella, Johnsonella, Actinobacillus, Abiotrophia* and an unassigned genus from family *Comamonadaceae* that had incidence high enough to reach statistical significance when compared to stool. One genus, *Mycoplasma,* had statistically higher abundance in visually-normal

tissue. The remaining 7 genera (including *Helicobacter*) hence fell to the no-difference category (see below). Within the tumour genera, 52 genera were significantly more abundant also in visually normal mucosa compared to stool, we call these mucosa genera. Additionally, 16 of tumour genera were significantly more abundant in tumour mucosa also compared to visually normal mucosa - we will call these tumour-specific genera.

51 genera were significantly depleted in tumour samples, compared to stool, of which 49 were also depleted in normal mucosa samples compared to stool (we call the latter stool genera).

156 genera showed no statistically significant differences in abundance between stool and tumours. Of these, 143 showed no difference between any of the sample types (the no difference category of genera).

1.3. The Landscape of Tumour Microbiome

For the description of tumour mucosa microbial heterogeneity, we only considered species that were statistically significantly more abundant in tumour mucosa compared to stool, to remove possible stool microbial contaminants on the tissue. We hence investigated the group of 57 tumour genera with special focus on the subgroup of 16 tumour-specific genera (*Gemella, Granulicatella, Parvimonas, Hungatella, Peptoclostridium, Flavonifractor, Selenomonas 3, Fusobacterium, Leptotrichia, Eikenella, Campylobacter, Slackia, Streptococcus, Howardella, Solobacterium, Defluviitaleaceae UCG-011)* (Figure 2A main manuscript). Table S5 details the closest species hits in QIIME and BLAST search for the ASVs representing these genera and summarises the state-of-the-art information about the association of the genera and their species with CRC.

The most common tumour genera (present in >50% tumour mucosa samples) were *Lachnoclostridium* and *Bacteroides* (detected on all tumours except one), followed by *Escherichia-Shigella* (93.3%) and *Fusobacterium* (88.8%), *Peptoclostridium* (80.3%), *Streptococcus* (79.2%), *Sutterella* (76.4%), *Haemophilus* (75.8%), *Flavonifractor* (73.0%), *Veillonella (70.7%)*, *Campylobacter* (66.3%), *Gemella* (63.5%), *Coprobacter* (59%), *Parvimonas* (56.7%) and uncultured genus from family *Lachnospiraceae* (95.5%). Even though commonly present across tumours, the median abundance of these genera in the samples ranged quite widely from 0.02% *(Parvimonas)* to 29.5% *(Bacteroides)* with median of 0.21%. It is to be emphasised that the mean abundance per genera in the case of 264 genera is 100/264 = 0.379%.

The least common tumour genera (present in less than <10% of tumour mucosa samples) were *Abiotrophia* (1.7%), *Prevotella 1* (2.3%), *Stomatobaculum* (2.8%), *Kingella* (3.4%), *Prevotellaceae UCG-003* (3.9%), *Phocaeicola* (4.5%), *Tatumella* (4.5%), *Johnsonella* (5.1%), *Shewenella* (5.1%), *Defluviitaleaceae UCG–011 (6.2%), Morganella* (7.3%) and unassigned genera from families *Comamonadaceae* (1.1%), *Peptostreptococcaceae* (3.4%) and *Veillonellaceae* (9.6%).

We further calculated on 127 triplets the sum of relative abundances (proportion) of the tumour genera and tumour-specific genera in each of the tumour sample types (Figure 2B main manuscript). The group of 57 tumour genera constituted 11% to 97% (median 59.6%) of tumour mucosa swab samples, 11.8% to 98.7% (median 58.7%) of visually normal swab samples and 1.5% to 89.9% (median 27.6%) of stool samples (Figure 2B main manuscript). In contrast, 18 tumour-specific genera represented between 0.0%–62.3% (median 3.1%) of genera found in individual tumour mucosa samples, 0.0%–42.6% (median 1.0%) of visually normal mucosa samples and 0.0%–23.1% (median 0.69%) of stools (Figure 2C main manuscript). In one patient, none of these genera was present in the tumour mucosa.

We closely inspected the tumour genera in the literature and found out that the category consists genera of predominantly oral bacteria (Table S10). *Slackia*, *Phocaeicola* and *Morganella* are periodontopathogen, *Gemella* and *Granulicatella* relate to endocarditis. *Aggregatibacter* and *Eikenella* are periodontopathogens associated with the endocarditis. *Gemella* and *Fusobacterium*, both presented in more than 50% of tumours, are associated with oral cancer. Moreover, *Solobacterium* and *Granulicatella* are more abundant in saliva of obese people.

1.4. Co-Occurence of Tumour Genera

To better understand the relationships between the 57 tumour genera (in contrast to analysis of cooccurence of one genus across sample types as described above), we performed microbial co-occurence analysis (Figure S4, Table S9). Overall, we observed 254 positive co-occurences and 14 negative co-occurences (in pairwise comparisons). The positive co-occurences were observed between most of the 16 tumour-specific genera (*Eikenella, Campylobacter, Solobacterium, Gemella, Leptotrichia, Parvimonas, Peptoclostridium, Howardella, Slackia, Selenomonas 3, Granulicatella* and *Fusobacterium)* and *Aggregatibacter, Alloprevotella, Treponema 2, Streptococcus, Selenomonas, Selenomonas 4, Senegalimassilia* and *Veilonella.*

Negative co-occurences were observed between *Selenomonas* and *Tyzzerella 4*, *Selenomonas* and *Massilias*; between *Selenomonas 3* and *Morganella*, between *Coprobacter* and *Treponema 2*, *Eggerthella* and *Peptostreptococcus*, *Coprobacter* and *Johnsonella*, *Coprobacter* and uncultured *Fusobacterium* sp.

When evaluating the results from a different perspective, all the identified genera from the phylum *Fusobacteria* (3 of 3) and *Spirochaetae* (1 of 1) belonged to the tumour genera and phylum *Proteobacteria* contained almost twice as much tumour genera as would be expected by chance (16 compared to 6 out of 34). None of the tumour genera belonged to *Euryarchaeota* (0 of 2), *Lentisphaerae* (0 of 1), *Tenericutes* (0 of 4) or *Verrucomicrobia* (0 of 2), *Deinococcus-Thermus* (0 of 1) or *Synergistetes* (0 of 4).

1.5. Microbiome and Clinical Variables

To characterize the overall heterogeneity of microbiome in CRC, we performed β-diversity analysis by NMDS (Figure S5, Figure S6). The results showed that tumour localisation is the factor with the highest influence on total microbiome composition for all sample types, explaining 3.1%, 2.7% and 2.3% of dispersion in Aitchison distance ($p < 0.05$) between samples, for stool, tumour and visually normal mucosa, respectively).

Additionally, tumour histological grade was shown to be linked with alteration of mucosa microbial composition, both for tumours and visually normal mucosa (R2 = 1% and 0.5%, respectively, $p < 0.05$), but not for stool samples (R2 = 0.7%, $p > 0.05$) (Table S11). According to the NMDS loading plots (Figure S5, Figure S6), tumour and visually normal mucosa microbiome consist of relatively similar list of microbial genera including *Fusobacterium*, *Solobacterium*, *Selenomonas 4* and *Dialister* "driving" the communities to advanced grade and right-side tumour localisation, whereas for stool community this trend was mostly absent.

We did not find significant associations between beta-diversity and tumour TNM staging for any sample type, but T separately was identified to be linked with visually-normal mucosa microbial community structure, respectively (Table S11). Among other clinical parameters, gender was found to be associated with tumour tissue microbiomes and patients' age was significantly linked with visually-normal tissue samples and stool microbiome (Table S11).

Text S2: Validation of Results on Publicly Available Data

We performed partial validation of our results of associations of microbiome with clinical variables on three publicly available datasets.

2.1. Validation of Tumour Microbiome Association with Tumour Localisation

The association of tumour microbiome with tumour localisation was validated on the dataset of Dajea et al. [1]. In the dataset was 23 tumour samples of which 12 were right-sided and 11 were left-sided. No grade information was provided, hence the validation did not use the grade*localisation interaction term. Additionally, our study uses three localisation categories: right-sided+transverse, left-sided and rectosigmoideum+rectum.

There are important differences, that have to be mentioned, in the preprocessing of the samples and the sequencing method that is far less sensitive are to Illumina with much lower read depth (range 7058–36975, with median 9830). To process available fastq files was used QIIME pipeline [2]

with the settings for 454 pyrosequencing. OTUs (Operational Taxonomic Units) were constructed using QIIME 1.9.1. Chimeras were detected with UCHIME in USEARCH v6.1.544 [3] and excluded from the further analysis. Taxonomy was assigned to each OTU with UCLUST algorithm and SILVA 123 was used as a reference database [4].

For statistical analysis was used exactly the same process as on our data. Zero multiplicative replacement [5] was applied prior to the centred log-ratio (clr) transformation [6]. On the normalised data was applied rank regression (R package Rfit [7]) to define differences between left-sided and right-sided tumours.

We were able to validate the association of the increased abundance of C*hristensenellaceae R-7* group in left-sided tumours (p = 0.0047) (Table 1, Table S13).

Table S1. Results of validation of associations of microbiome with tumour location on the Dejea et al. [1] dataset.

Genera	Intercept	Effect Right vs. Left	Right vs. Left (p-Value)	Right vs. Left (Adjusted p-Value)	Drop Test p-Value	Drop Test Adjusted p-Value
Christensenellaceae R-7 group	2.05	−2.57	0.00005	0.00062	0.00467	0.06067
Holdemania	−0.79	0.39	0.20323	0.88068	0.18636	0.80756
Family XIII AD3011 group	0.28	−0.75	0.11893	0.77306	0.13830	0.80756
Selenomonas 4	−0.80	0.09	0.72013	0.96985	0.54683	0.84305
Clostridium sensu stricto 1	−0.62	0.14	0.59663	0.96985	0.50443	0.84305
Alloprevotella	−0.55	−0.18	0.78831	0.96985	0.64850	0.84305
Fusicatenibacter	1.50	0.64	0.52884	0.96985	0.50011	0.84305
Alloprevotella	−0.55	−0.18	0.78831	0.96985	0.64850	0.84305
Oribacterium	−0.80	0.07	0.57976	0.96985	0.49294	0.84305
Fretibacterium	−0.80	0.08	0.74902	0.96985	0.59758	0.84305
Campylobacter	−0.91	−0.10	0.82064	0.96985	0.81950	0.96850
Ruminococcaceae UCG-013	−0.73	0.00	1.00000	1.00000	1.00000	1.00000
Coprococcus 1	1.80	0.00	1.00000	1.00000	1.00000	1.00000

2.2. Validation of stool microbiome association with pTNM staging and AJCC staging

The association of stool microbiome with AJCC staging and TNM staging was validated in two datasets (Zeller et al. [8] and Feng et al. [9]). The processed datasets with taxonomic information were used as available in R package curatedMetagenomicData [10] and were normalised using the clr transformation before the analysis.

All associations were tested using rank regression (R package Rfit [7]). The dataset of Feng et al. only contained one M1 sample; hence we only analysed associations with AJCC staging, T stage and N stage.

We validated the decrease of *Ruminococcaceae* in the stool of patients with higher pT stage (p = 0.00113) and decrease of *Dorea* in the stool of patients with local lymphnode metastases (p = 0.00011) (Table 2, Table S13).

Table S2. Results of validation of associations of microbiome with tumour location on the Dejea et al., Zeller et al. and Feng et al. datasets [1,8,9]. (pT—tumour pathologic, pN—node pathologic, pM—synchronous distant metastasis)

Clinical Variable	Genera	Zeller et al. [8]		Feng et al. [9]	
		**Effect pT3–4 vs pTis-2**	**pT3–4 vs pTis-2 (*p*-Value)**	**Effect pT3–4 vs pTis-2**	**pT3–4 vs pTis-2 (*p*-Value)**
**pT stage**	*Escherichia*	−0.69	0.22003	−0.15	0.87368
	Prevotella	−0.26	0.74270	−1.47	0.14582
	Ruminococcaceae_noname	−0.60	0.27940	−3.03	0.00113
**pN stage**		**Zeller et al. [8]**		**Feng et al. [9]**	
	Genera	**Effect pN1–2 vs pN0**	**effect pN1–2 vs pN0 (*p*-value)**	**Effect pN1–2 vs pN0**	**effect pN1–2 vs pN0 (*p*-Value)**
	Streptococcus	0.26	0.47568	−0.41	0.46552
	Faecalibacterium	−0.05	0.85487	0.05	0.93137
	Dorea	−0.29	0.35578	−1.60	0.00011
	Lachnospiraceae_noname	−0.09	0.71529	−0.81	0.08644
	Akkermansia	−0.27	0.73794	0.15	0.89882
	Porphyromonas	0.05	0.88648	0.63	0.51784
	Campylobacter	0.01	0.95360	0.17	0.57111
**pM stage**		**Zeller et al. [8]**			
	Genera	**Effect pM1 vs pM0**	**Effect pM1 vs pM0 (*p*-value)**		
	Coprococcus	−0.45	0.19097		
	Akkermansia	0.44	0.61293		
	Solobacterium	−1.13	0.07450		
	Ruminococcaceae_noname	0.22	0.70928		
**AJCC stage**		**Zeller et al. [8]**		**Feng et al. [9]**	
	Genera	**Effect III–IV vs 0–II**	**Effect III–IV vs 0–II (*p*-Value)**	**Effect III–IV vs 0–II**	**Effect III–IV vs 0–II (*p*-Value)**
	Akkermansia	0.69	0.39792	0.15	0.89882

References

1. Dejea, C.M.; Wick, E.C.; Hechenbleikner, E.M.; White, J.R.; Mark Welch, J.L.; Rossetti, B.J.; Peterson, S.N.; Snesrud, E.C.; Borisy, G.G.; Lazarev, M.; et al. Microbiota Organization Is a Distinct Feature of Proximal Colorectal Cancers. *Proc Natl Acad Sci U S A* **2014**, *111*, 18321–18326, doi:10.1073/pnas.1406199111.
2. Caporaso, J.G.; Kuczynski, J.; Stombaugh, J.; Bittinger, K.; Bushman, F.D.; Costello, E.K.; Fierer, N.; Peña, A.G.; Goodrich, J.K.; Gordon, J.I.; et al. QIIME Allows Analysis of High-Throughput Community Sequencing Data. *Nat Methods* **2010**, *7*, 335–336, doi:10.1038/nmeth.f.303.
3. Edgar, R.C.; Haas, B.J.; Clemente, J.C.; Quince, C. and Knight, R. UCHIME improves sensitivity and speed of chimera detection. *Bioinform* **2011**, *27*, 2194–2200. doi: 10.1093/bioinformatics/btr381
4. Pruesse, E.; Quast, C.; Knittel, K.; Fuchs, B.M.; Ludwig, W.; Peplies, J.; Glöckner, F.O. SILVA: A Comprehensive Online Resource for Quality Checked and Aligned Ribosomal RNA Sequence Data Compatible with ARB. *Nucleic Acids Res* **2007**, *35*, 7188–7196, doi:10.1093/nar/gkm864.
5. Martín-Fernández, J.-A.; Hron, K.; Templ, M.; Filzmoser, P.; Palarea-Albaladejo, J. Bayesian-Multiplicative Treatment of Count Zeros in Compositional Data Sets. *Statistical Modelling: An International Journal* **2015**, *15*, 134–158, doi:10.1177/1471082X14535524.
6. Aitchison, J. The Statistical Analysis of Compositional Data. *Journal of the Royal Statistical Society: Series B (Methodological)* **1982**, *44*, 139–160, doi:10.1111/j.2517-6161.1982.tb01195.x.
7. Kloke, J.D.; McKean, J.W. Rfit: Rank-Based Estimation for Linear Models. *The R journal* **2012**, *4*.
8. Zeller, G.; Tap, J.; Voigt, A.Y.; Sunagawa, S.; Kultima, J.R.; Costea, P.I.; Amiot, A.; Böhm, J.; Brunetti, F.; Habermann, N.; et al. Potential of Fecal Microbiota for Early-Stage Detection of Colorectal Cancer. *Mol. Syst. Biol.* **2014**, *10*, 766, doi:10.15252/msb.20145645.
9. Feng, Q.; Liang, S.; Jia, H.; Stadlmayr, A.; Tang, L.; Lan, Z.; Zhang, D.; Xia, H.; Xu, X.; Jie, Z.; et al. Gut Microbiome Development along the Colorectal Adenoma-Carcinoma Sequence. *Nat Commun* **2015**, *6*, 6528, doi:10.1038/ncomms7528.

10. Pasolli, E.; Schiffer, L.; Manghi, P.; Renson, A.; Obenchain, V.; Truong, D.T.; Beghini, F.; Malik, F.; Ramos, M.; Dowd, J.B.; et al. Accessible, Curated Metagenomic Data through ExperimentHub. *Nat Methods* **2017**, *14*, 1023–1024, doi:10.1038/nmeth.4468.

© 2021 by the authors. Licensee MDPI, Basel, Switzerland. This article is an open access article distributed under the terms and conditions of the Creative Commons Attribution (CC BY) license (http://creativecommons.org/licenses/by/4.0/).
