# Supplementary figures and images for: Colorectal Tumour Mucosa Microbiome Is Enriched in Oral Pathogens and Defines Three Subtypes That Correlate with Markers of Tumour Progression
Source: Cancers (Basel). 2021 Sep 25;13(19):4799. doi: 10.3390/cancers13194799 (PMC8507728; doi:10.3390/cancers13194799)

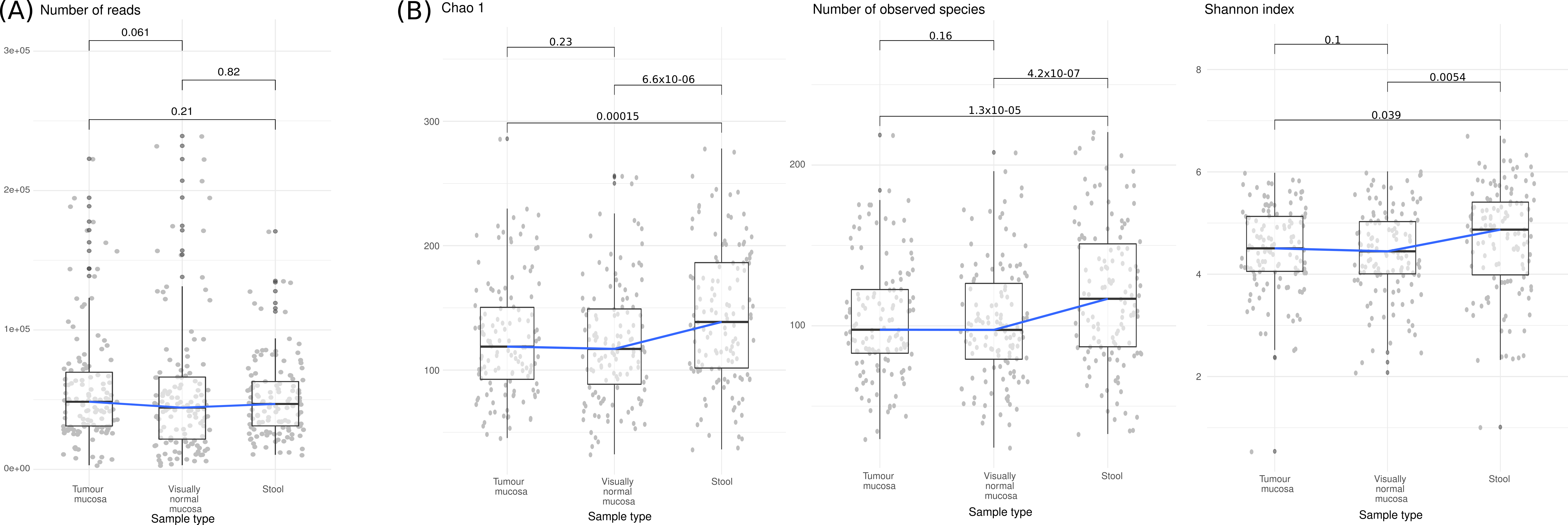

Supplement: Supplementary file 1 [file cancers-13-04799-s001.zip › cancers-1377747-supplementary-updated final/Supplementary Figures/FigS1.png]

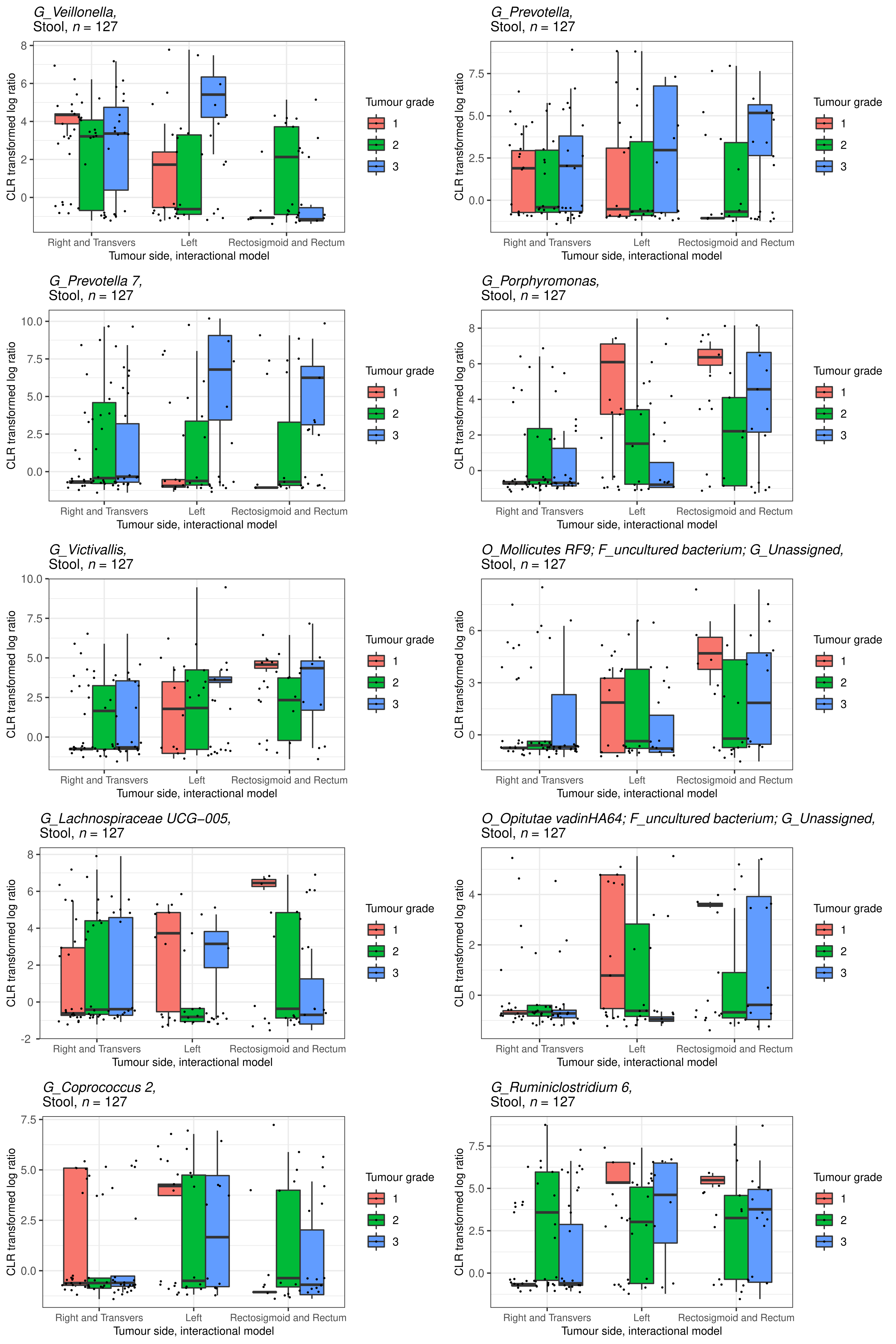

Supplement: Supplementary file 1 [file cancers-13-04799-s001.zip › cancers-1377747-supplementary-updated final/Supplementary Figures/FigS10.png]

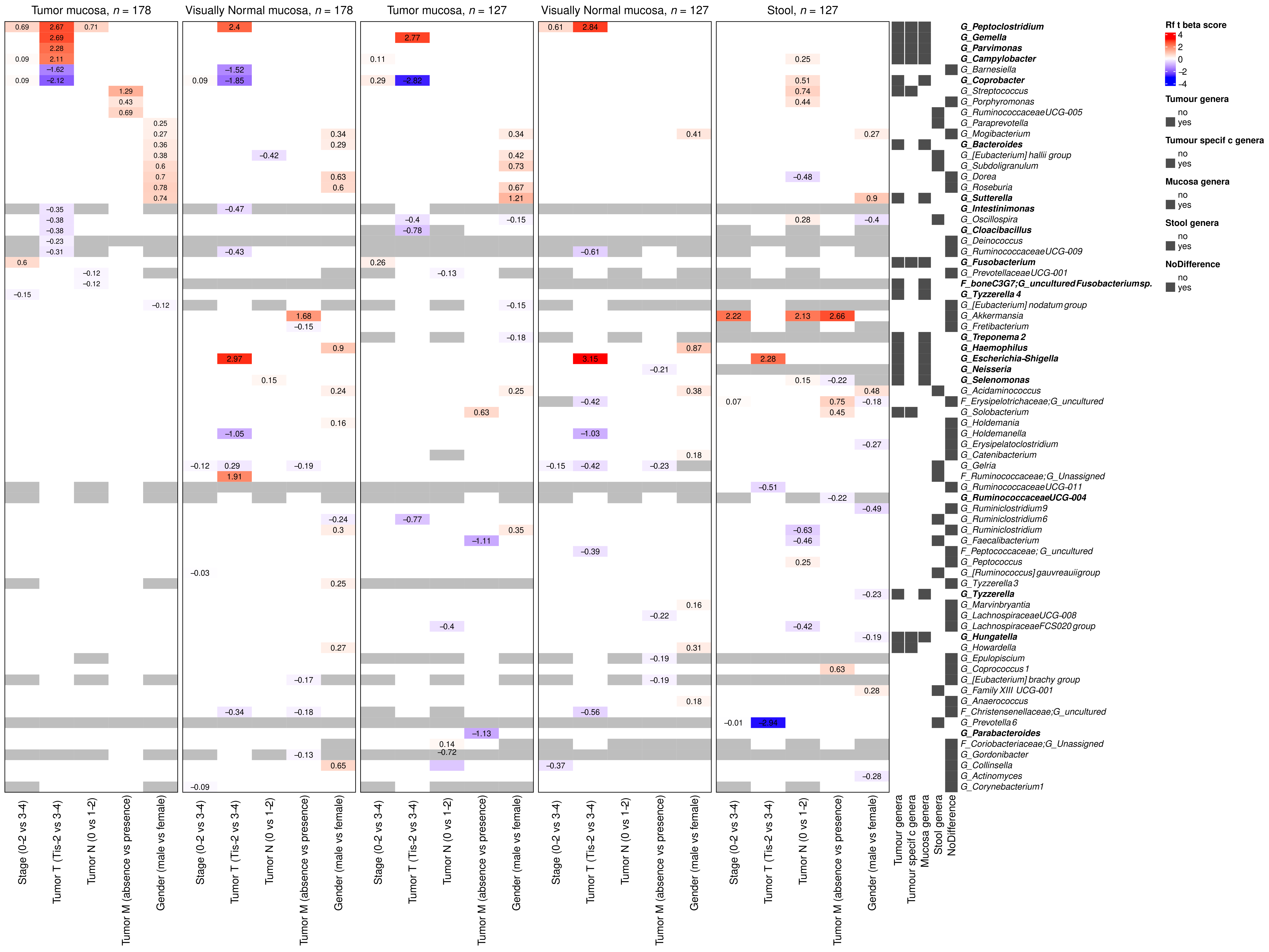

Supplement: Supplementary file 1 [file cancers-13-04799-s001.zip › cancers-1377747-supplementary-updated final/Supplementary Figures/FigS11.png]

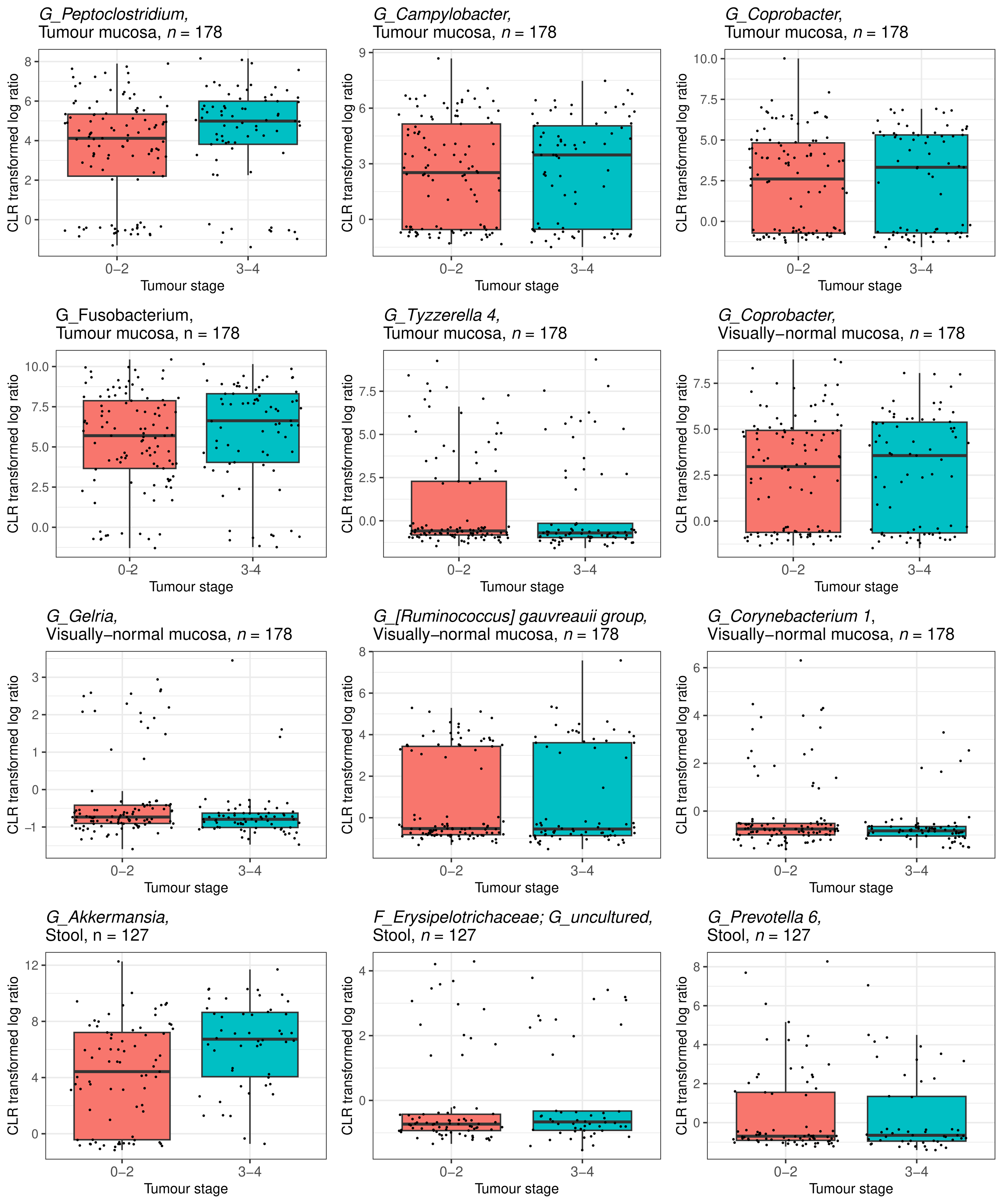

Supplement: Supplementary file 1 [file cancers-13-04799-s001.zip › cancers-1377747-supplementary-updated final/Supplementary Figures/FigS12.png]

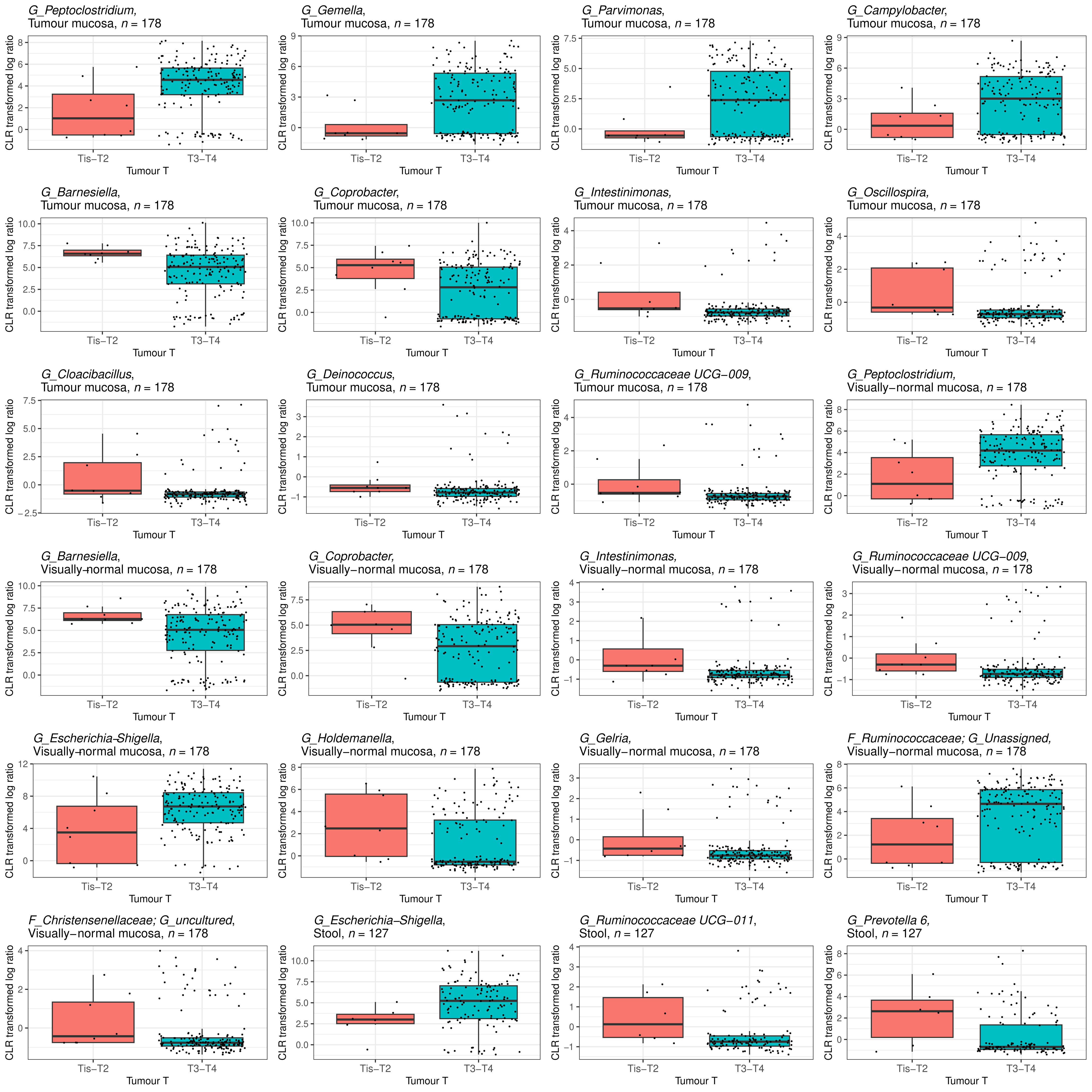

Supplement: Supplementary file 1 [file cancers-13-04799-s001.zip › cancers-1377747-supplementary-updated final/Supplementary Figures/FigS13.png]

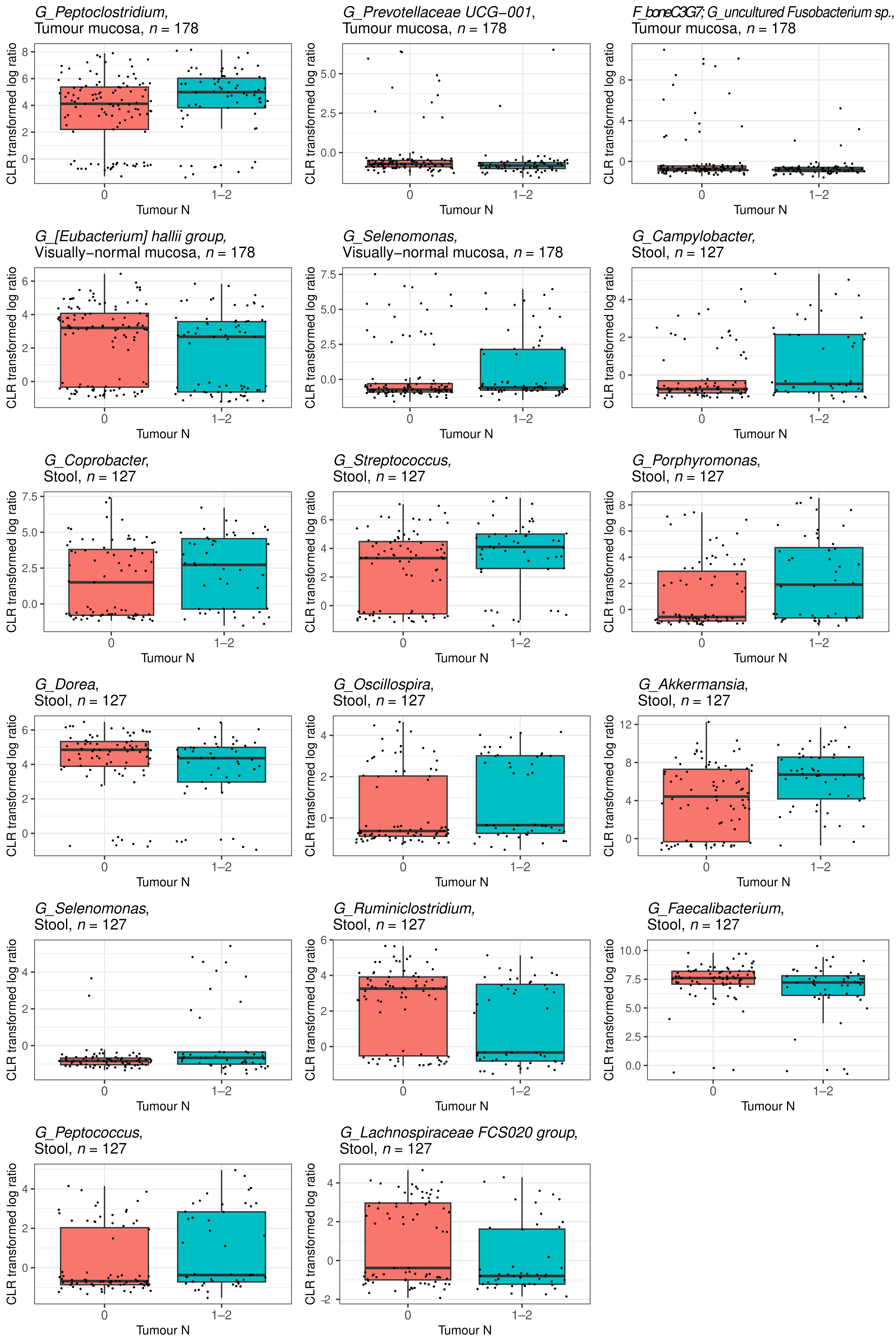

Supplement: Supplementary file 1 [file cancers-13-04799-s001.zip › cancers-1377747-supplementary-updated final/Supplementary Figures/FigS14.png]

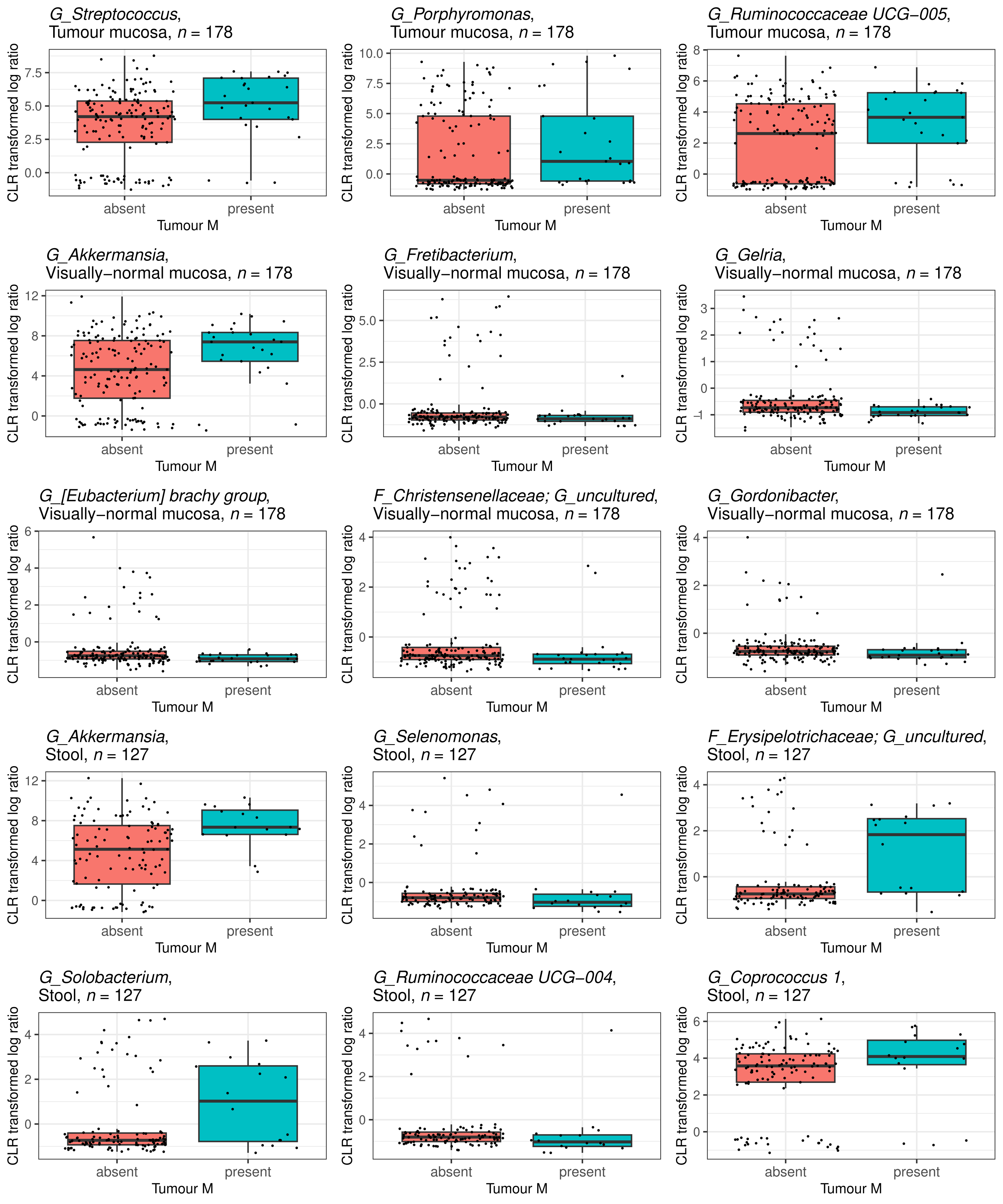

Supplement: Supplementary file 1 [file cancers-13-04799-s001.zip › cancers-1377747-supplementary-updated final/Supplementary Figures/FigS15.png]

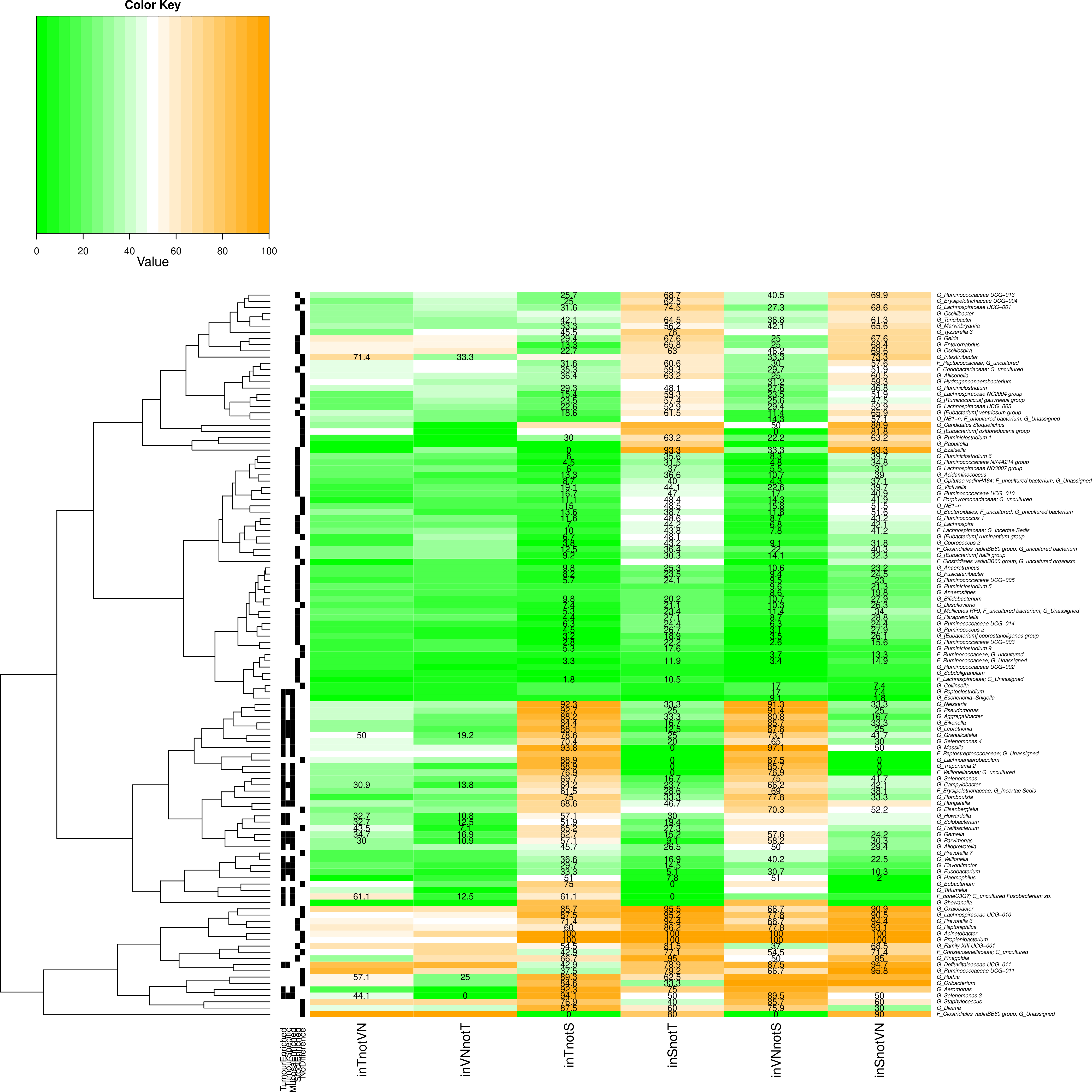

Supplement: Supplementary file 1 [file cancers-13-04799-s001.zip › cancers-1377747-supplementary-updated final/Supplementary Figures/FigS16.png]

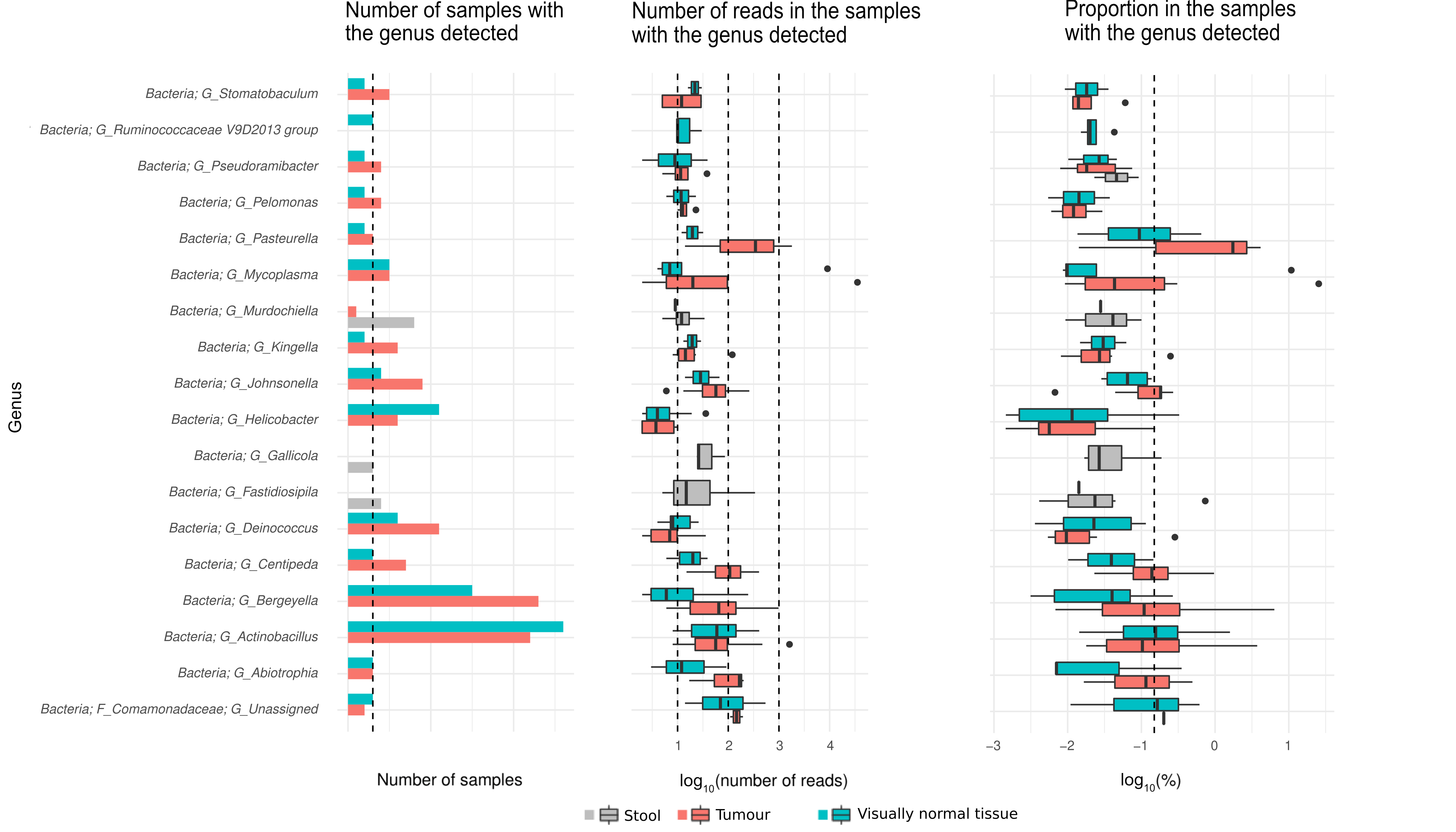

Supplement: Supplementary file 1 [file cancers-13-04799-s001.zip › cancers-1377747-supplementary-updated final/Supplementary Figures/FigS2.png]

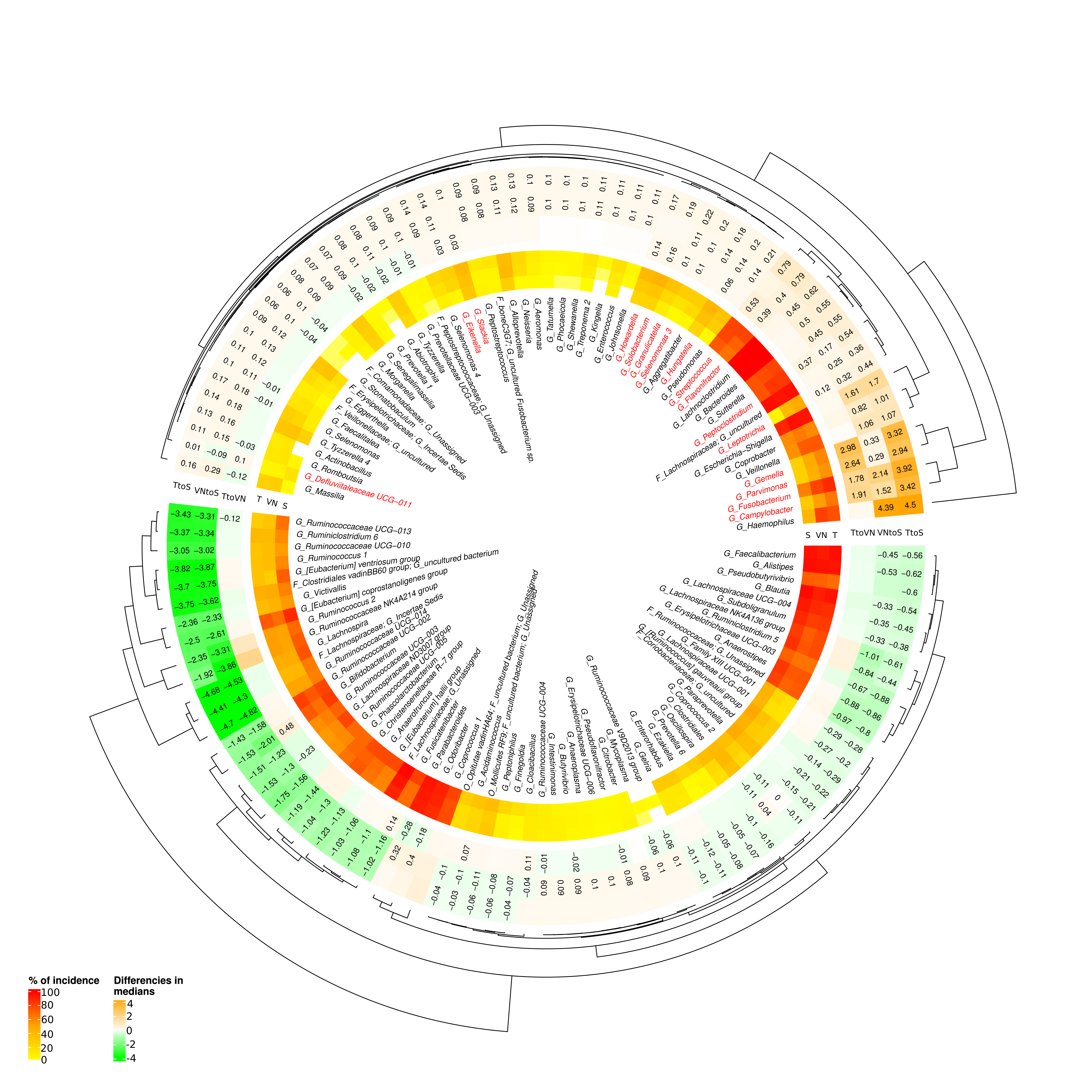

Supplement: Supplementary file 1 [file cancers-13-04799-s001.zip › cancers-1377747-supplementary-updated final/Supplementary Figures/FigS3.png]

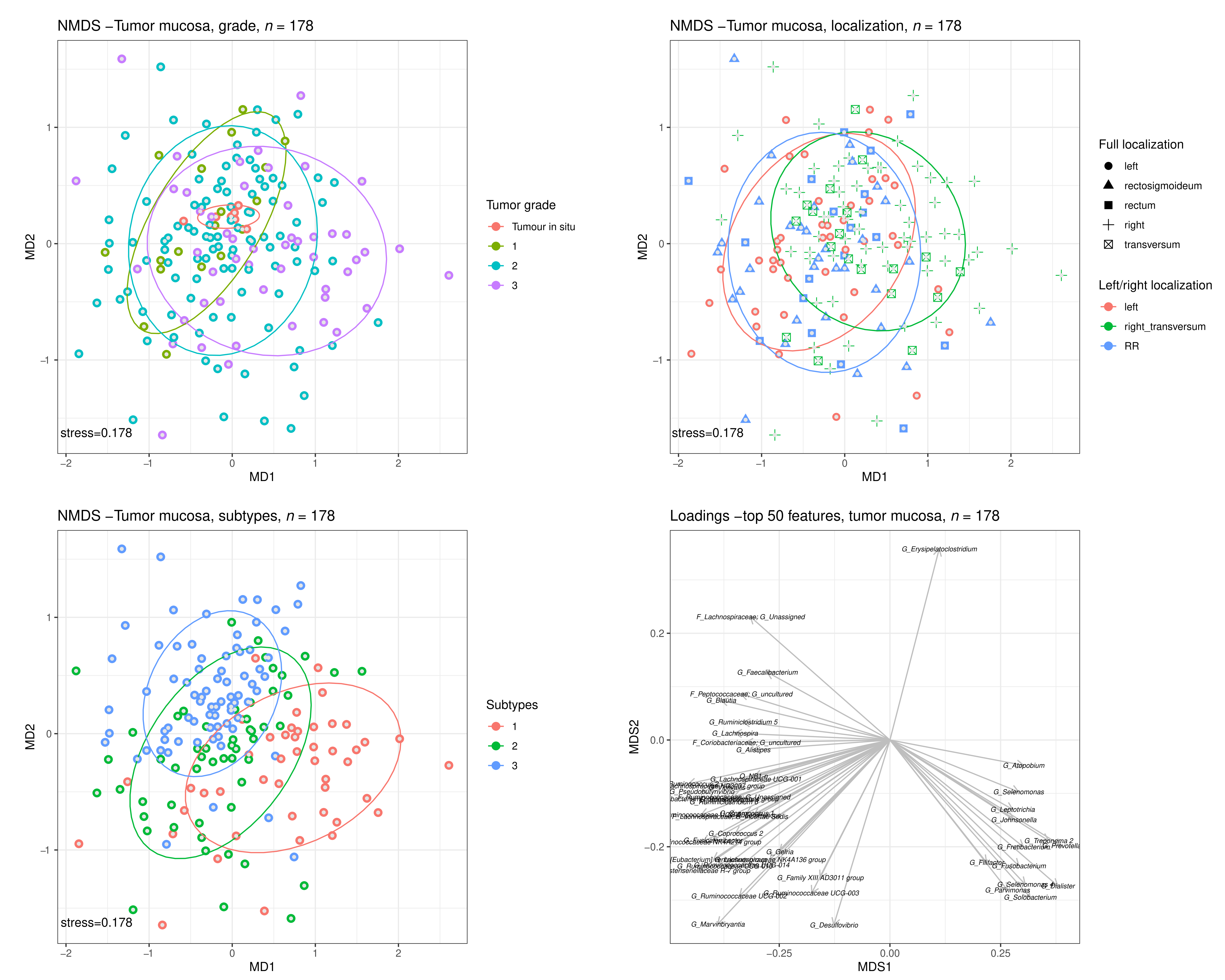

Supplement: Supplementary file 1 [file cancers-13-04799-s001.zip › cancers-1377747-supplementary-updated final/Supplementary Figures/FigS5.png]

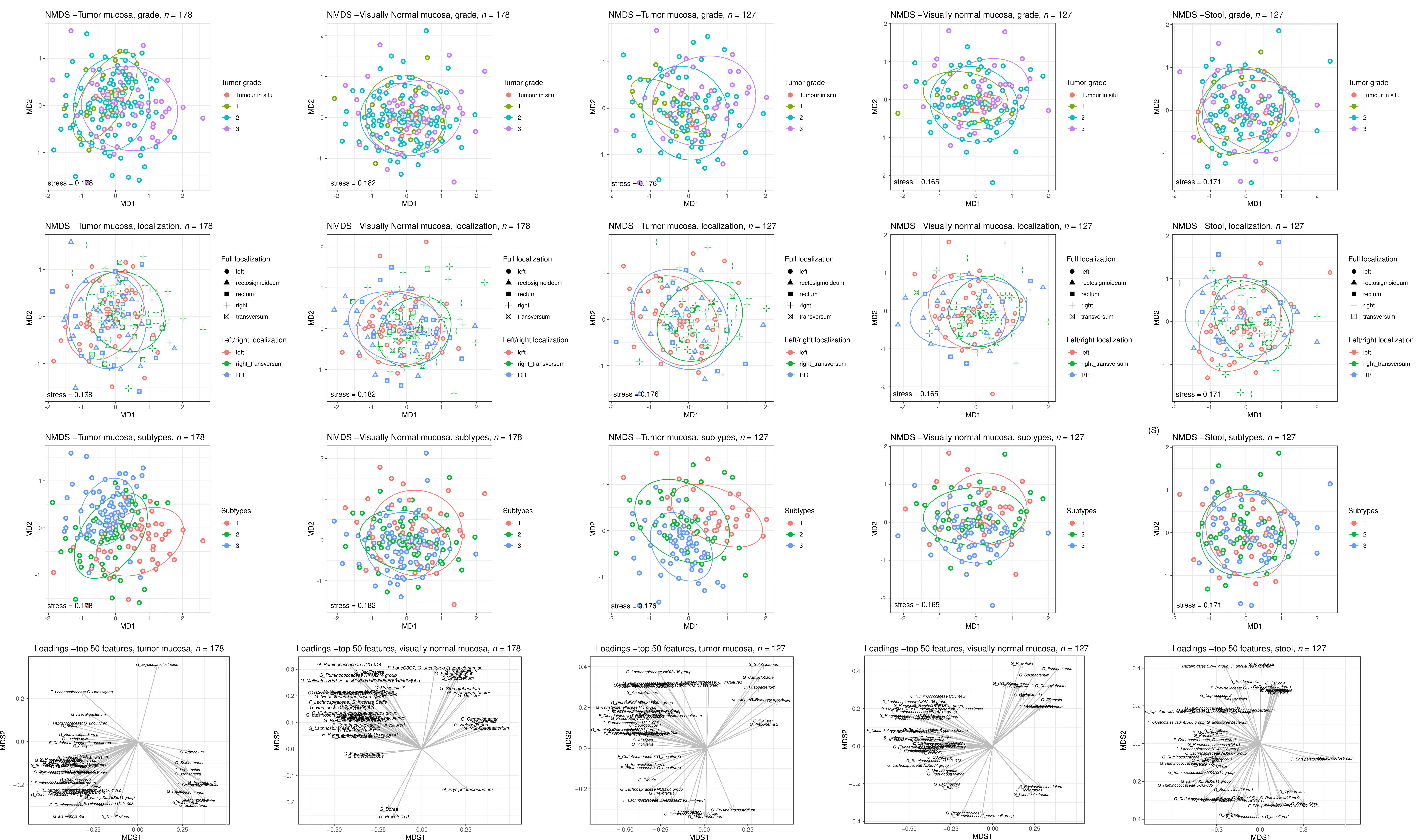

Supplement: Supplementary file 1 [file cancers-13-04799-s001.zip › cancers-1377747-supplementary-updated final/Supplementary Figures/FigS6.png]

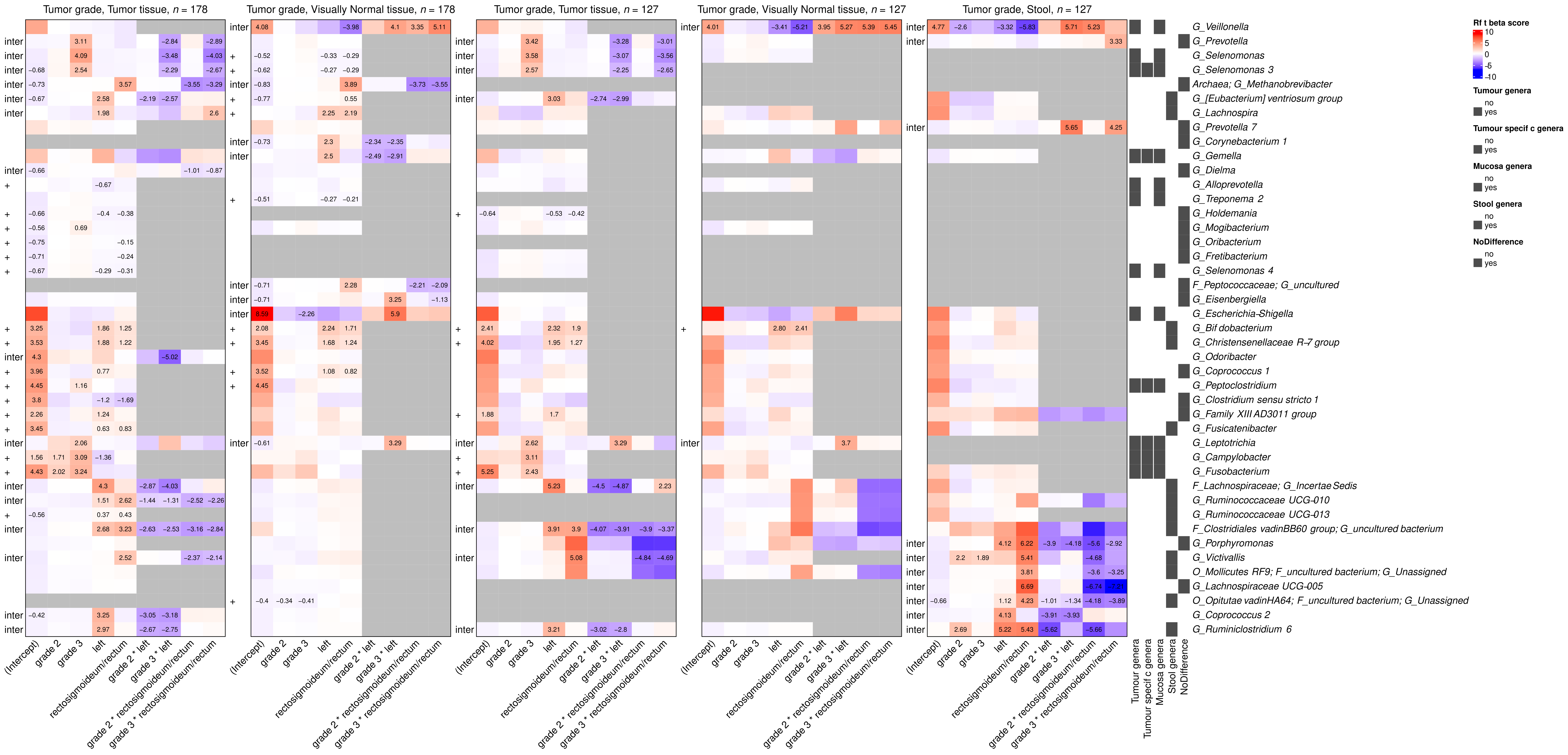

Supplement: Supplementary file 1 [file cancers-13-04799-s001.zip › cancers-1377747-supplementary-updated final/Supplementary Figures/FigS7.png]

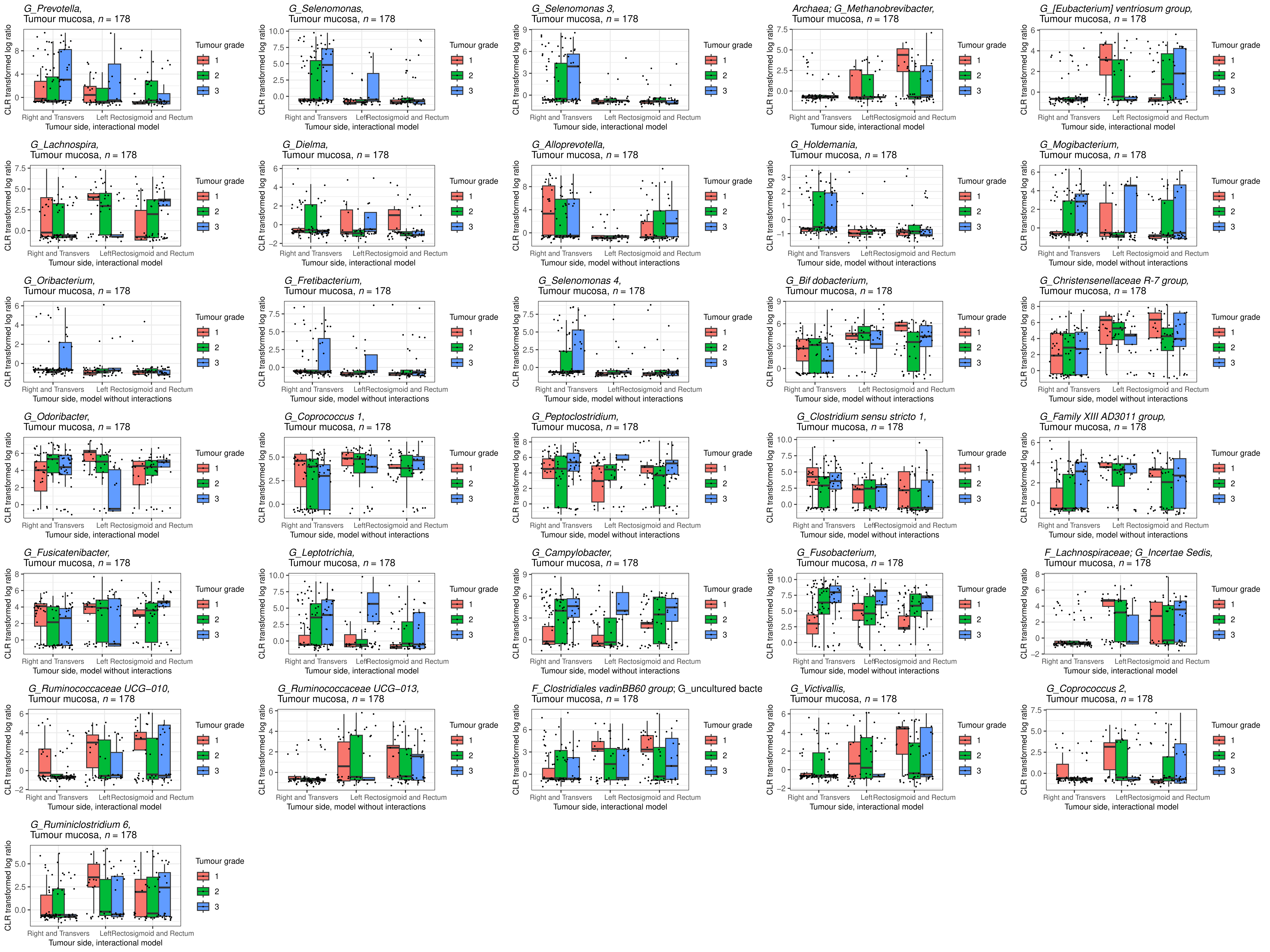

Supplement: Supplementary file 1 [file cancers-13-04799-s001.zip › cancers-1377747-supplementary-updated final/Supplementary Figures/FigS8.png]

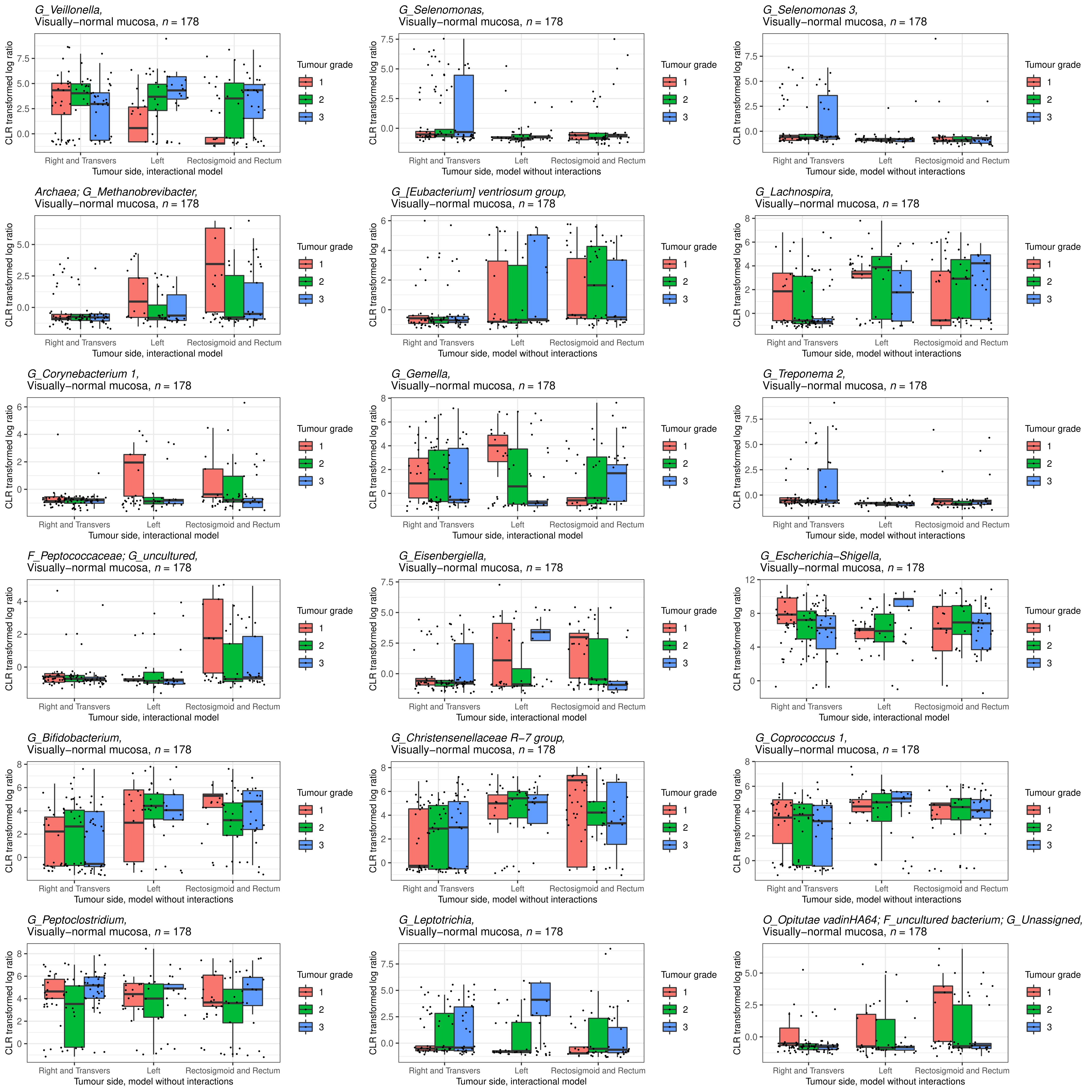

Supplement: Supplementary file 1 [file cancers-13-04799-s001.zip › cancers-1377747-supplementary-updated final/Supplementary Figures/FigS9.png]
